# Two OB-fold proteins from a Gram-positive conjugative element engage in relaxosome assembly and DNA processing

**DOI:** 10.1093/nar/gkaf1161

**Published:** 2025-11-13

**Authors:** Haifa Laroussi, Robine Maffo-Woulefack, Julien Cappèle, Hicham Sekkouri Alaoui, Lysiane Lessure, Maha Zahaf, Jean-Michel Girardet, Emilie Clément, Louise Thiriet, Stéphane Bertin, Dunkan Begue, Badreddine Douzi, Yvonne Roussel, Claude Didierjean, Frédérique Favier, Pascale Tsan, Nathalie Leblond-Bourget, Nicolas Soler

**Affiliations:** Université de Lorraine, INRAE, DynAMic, F-54000 Nancy, France; Université de Lorraine, INRAE, DynAMic, F-54000 Nancy, France; Universite de Lorraine, CNRS, CRM2, F-54000 Nancy, France; Université de Lorraine, INRAE, DynAMic, F-54000 Nancy, France; Universite de Lorraine, CNRS, CRM2, F-54000 Nancy, France; Université de Lorraine, INRAE, DynAMic, F-54000 Nancy, France; Université de Lorraine, INRAE, DynAMic, F-54000 Nancy, France; Universite de Lorraine, INRAE, IAM, F-54000 Nancy, France; Universite de Lorraine, INRAE, IAM, F-54000 Nancy, France; Université de Lorraine, INRAE, DynAMic, F-54000 Nancy, France; Université de Lorraine, INRAE, DynAMic, F-54000 Nancy, France; Universite de Lorraine, CNRS, CRM2, F-54000 Nancy, France; Université de Lorraine, INRAE, DynAMic, F-54000 Nancy, France; Université de Lorraine, INRAE, DynAMic, F-54000 Nancy, France; Universite de Lorraine, CNRS, CRM2, F-54000 Nancy, France; Universite de Lorraine, CNRS, CRM2, F-54000 Nancy, France; Universite de Lorraine, CNRS, CRM2, F-54000 Nancy, France; Université de Lorraine, INRAE, DynAMic, F-54000 Nancy, France; Université de Lorraine, INRAE, DynAMic, F-54000 Nancy, France

## Abstract

Integrative and conjugative elements (ICEs) are widespread genomic islands that propagate by bacterial conjugation. ICEs often spread antibiotic resistance genes and other adaptive genes conferring evolutionary advantages to their hosts. At the initiation of conjugation, the ICE DNA is processed by the relaxase, a transesterase that recognises its origin-of-transfer. ICE*St3*/Tn*916*/ICE*Bs1* superfamily of ICEs is widespread in Gram-positive bacteria, and it encodes uncanonical relaxases classified within the unique MOB_T_ family. This study elucidates the roles of OrfL and OrfM, two OB-fold proteins from ICE*St3*, in its conjugative transfer. We demonstrated that OrfL and OrfM form a complex and function as relaxosomal auxiliary proteins with RelSt3, the ICE*St3* MOB_T_ relaxase. Through conjugation assays, we established that they are both essential for ICE*St3* conjugation. The NMR 3D structure of OrfM revealed an OB-fold protein with several flexible regions. Our results also suggest a networking role for OrfL as it was shown by BACTH to interact with both the relaxase and with the cellular PcrA helicase involved in rolling-circle replication of mobile genetic elements in Firmicutes. Our data provide a description of essential participation of OB-fold proteins in a relaxosome, contrasting with most of other known relaxosomes in both Gram-negative and Gram-positive bacteria, which primarily involve ribbon–helix–helix proteins.

## Graphical Abstract

**Graphical Abstract:**
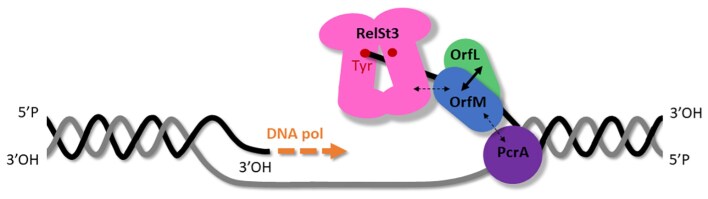

## Introduction

Bacterial genomes evolve mainly through horizontal gene transfer, with conjugation being one of the major evolution mechanisms [1]. Conjugation requires cell-to-cell contact between a donor and a recipient cell [2]. It is mediated by conjugative elements, including conjugative plasmids and integrative and conjugative elements (ICEs) [3, 4]. In addition to their transfer function, conjugative elements encode other functions that may provide an adaptive advantage to their host, such as resistance to antibiotics. Indeed, the massive use of antibiotics led to a significant increase in the prevalence of antibiotic-resistant genes in bacterial genomes, largely due to the transfer of conjugative elements carrying these genes [5, 6]. Addressing this major public health issue requires a thorough understanding of the mechanisms of conjugative transfer to design strategies aimed at limiting their dissemination.

The molecular mechanism of bacterial conjugation involves two major steps [7]. First, the DNA to be transferred is processed by the relaxosome complex [8]. Second, the DNA is transferred from the donor to the recipient cell through a type-IV secretion system (T4SS), a large multiprotein complex that spans the bacterial cell envelope [9]. The key actor of the relaxosome complex is the relaxase, a transesterase that recognises a specific sequence on the conjugative element, the origin of transfer (*oriT*) [8]. The relaxase nicks the *oriT* and remains covalently attached to the 5′-end, whereas the 3′-end is used to initiate Rolling Circle Replication (RCR). In the recipient cell, the transferred DNA is thought to be recircularised through a phosphodiesterification step catalysed by the relaxase. Subsequently, the complementary strand DNA is synthesised by the host DNA replication machinery [5, 10]. Besides the relaxase, relaxosomes often include small auxiliary proteins, which assist the relaxase in its functions, such as binding to the *oriT* and/or stimulating its nicking activity [8, 11, 12]. In most relaxosomes studied so far, auxiliary proteins harbour a Ribbon–Helix–Helix (RHH) domain, whose function is to bind DNA in a sequence-specific manner [13, 14]. Among the best characteried auxiliary proteins are the TraM and TraY proteins encoded by the *Escherichia coli* F plasmid [8], both harbouring RHH domains. They were shown to bind specific sequences on *oriT*, triggering DNA bending and therefore facilitating relaxase binding on *oriT*. Additional examples of RHH relaxosomal proteins are the TrwA protein from the R388 plasmid in *E. coli* [15], or in Gram-positive bacteria, the PcfF protein from the pCF10 plasmid in *Enterococcus faecalis* [12], and the two auxiliary proteins from the pLS20 plasmid in *Bacillus subtilis* (Aux1_LS20_ and Aux2_LS20_) [16]. Very few auxiliary proteins from ICEs have been studied to date. Rare examples include the MobC protein (also harbouring an RHH domain) from Tn*1549* of *Enterococci* [17]and the HelP protein from ICE*Bs1* (see below) [18].

The Tn*916* superfamily is widespread in Gram-positive bacteria and especially in Firmicutes [19]. It encompasses several families of ICEs represented by the following study models: Tn*916* from *E. faecalis* [20], ICE*Bs1* from *B. subtilis* [21], and ICE*St3* from *Streptococcus thermophilus* [22]. Most of the Tn*916*-related elements are responsible for the spread of antimicrobial resistance genes (especially resistance to tetracycline, macrolides, and bacitracin), even beyond the Firmicutes phylum [20, 23].

ICEs of this Tn*916*/ICE*Bs1*/ICE*St3* superfamily all encode non-canonical relaxases belonging to the unique MOB_T_ family. These MOB_T_ relaxases are related to *Rep_trans* RCR initiators, which distinguishes them from the canonical relaxase families belonging to HUH endonucleases [10, 24–26]. We recently characterised the DNA processing steps orchestrated by the MOB_T_ relaxase encoded by ICE*St3*, named RelSt3 [27]. We demonstrated that RelSt3 is able to recognise a *bind* site that is distantly located from its *nic* site, in contrast to most known systems where the *bind* site is usually closely located from the *nic* site. While the *nic* site sequence is well conserved across the Tn*916*/ICE*Bs1*/ICE*St3* superfamily (and also with the replication origin of *Rep_trans* proteins), the *bind* site sequence seems to be less conserved.

The MOB_T_ relaxases were also demonstrated to enable autonomous RCR of the ICE, and this RCR is required for the maintenance of the element [28, 29]. Interestingly, ICE*Bs1* encodes a small protein, HelP (for helicase processivity), which plays a significant role in its transfer. HelP protein was shown to enhance the processivity of helicases of the superfamily I [18]. PcrA, a conserved cellular SFI helicase encoded by Firmicutes, has long been known to be involved in RCR [30, 31], and its processivity is supposed to be enhanced by HelP. Chromatin immunoprecipitation (ChIP) experiments revealed that HelP is associated with ICE*Bs1 oriT*, and this association is dependent on the nicking activity of NicK, the MOB_T_ relaxase encoded by ICE*Bs1*. Thomas and colleagues also noticed that the 3D structure of a HelP homologue, SAG0934 from *Streptococcus agalactiae*, displays an oligonucleotide/oligosaccharide binding (OB) fold [18]. OB-fold domains are commonly found in many proteins involved in a wide range of biological processes [32], many of them being involved in DNA replication and DNA repair in the three domains of life through their ability to interact with single-stranded DNA (ssDNA), double-stranded DNA (dsDNA), and/or proteins [33, 34].

We found that ICE*St3* encodes two HelP homologues, namely OrfL and OrfM proteins. In this work, we investigated the biological roles of these two proteins. Both *orfL* and *orfM* genes were shown to be essential for ICE*St3* conjugation. We demonstrated that they form a stable OrfL–OrfM complex. NMR structural analysis revealed an unusual OB-fold for OrfM, and *in silico* structural predictions confidently propose an OB-fold for OrfL and an OrfL–OrfM heterodimer. We therefore investigated the ability of OrL/OrfM proteins to interact with DNA, and with potential protein partners such as the RelSt3 relaxase and the PcrA helicase. Furthermore, we studied the effect of OrfL/OrfM proteins on the enzymatic activities of RelSt3. This HelP/OrfL/OrfM family of proteins constitutes a unique example of OB-fold proteins involved in conjugative relaxosomes.

## Materials and methods

### Bacterial strains and plasmids

The bacterial strains and plasmids used in this study are listed in Supplementary Tables S1 and S2, respectively. *Streptococcus thermophilus* LMG18311 harbouring ICE*St3*, labelled with a chloramphenicol resistance gene, or its derivative depleted of the *orfL* or *orfM* genes, was used as donor strain during mating experiments. A derivative strain from *S. thermophilus* LMG18311 that harbours a pMG36e plasmid carrying an erythromycin resistance gene was used as the recipient strain. *E. coli* EC101 was used as the host for cloning procedures with pG+host9. *E. coli* DH5α was used for cloning procedures and *E. coli* BL21 (*DE3*) was used for heterologous protein expression.

### Construction of *S. thermophilus* ICE*St3*Δ*orfL* and ICE*St3*Δ*orfM* strains and of the respective complemented strains

The depleted *S. thermophilus* ICE*St3*Δ*orfL* and *S. thermophilus* ICE*St3*Δ*orfM* strains were constructed using the thermosensitive pG^+^host9 plasmid, in a strategy similar to that used previously for the depletion of *orfJ* gene in [25], derived from [35]. The plasmid carrying 1 kb flanking regions upstream and downstream of the target genes was introduced into the cells. Transformants were selected at 30°C to facilitate homologous recombination at the chromosomal target site. Next, the integrant clones were grown at 42°C without plasmid selection to promote its loss. Plasmid-cured clones were screened by Polymerase Chain Reaction (PCR) to select mutants with the desired markerless deletion. To construct complemented strains, the CDS encoding OrfL and OrfM were amplified and assembled by Gibson assembly (NEBuilder^®^ HiFi DNA Assembly Master Mix) so that the CDS was placed under the control of a promotor inducible by anhydrotetracycline. The linear construct also included (i) a spectinomycin resistance gene transcribed downstream in the same operon and (ii) 1 kb flanking regions allowing integration of the cassette by recombination in the region of *S. thermophilus* LMG18311 genome close to the gene encoding tRNASer. The corresponding complemented strains were generated by introduction of the cassette by natural transformation into the *S. thermophilus* ICE*St3*Δ*orfL* and *S. thermophilus* ICE*St3*Δ*orfM* strains, respectively, as described in Maffo-Woulefack *et al.*, 2025 [36]. Briefly, 20 µl of overnight cultures of these mutant strains were mixed with 500 ng of linear DNA cassette in the presence of 250 nM of LPYFAGCL synthetic peptide to induce natural competence. Preheated milk was added to reach 200 µl final volume, and the mixture was incubated for 5 h at 42°C. Next, cells were plated on LM17 agar plates supplemented with spectinomycin (500 ng/µl) for selection of complemented strains. Mutant and complemented strains were checked by sequencing the corresponding region. The oligonucleotides used for these constructs are listed in Supplementary Table S3.

### Conjugation experiments

Conjugation experiments were carried out as described previously [37]. Briefly, *S. thermophilus* LMG18311-derived donor strains (containing ICE*St3*) and the recipient strain were grown overnight in the presence of the appropriate antibiotics (chloramphenicol 4 µg/ml for the donor strain, and erythromycin 5 µg/ml for the recipient strain). Overnight cultures were diluted 1:100 and further grown without any antibiotic. When cultures reached an OD_600nm_ of 0.4, donor and recipient cells were mixed (1:1 ratio) and concentrated 30-fold in LM17 broth. For each mating experiment, two 150 μl aliquots of cells were spread on 0.45 μm pore-size nitrocellulose filters (Millipore) deposited on soft agar (0.8%) LM17 plates and then incubated overnight at 42°C. Transconjugant (TC) cells were recovered from the filters with 10 ml of LM17, directly spread, or concentrated 10 times before plating onto LM17 plates containing chloramphenicol and erythromycin. After a 24 h incubation at 42°C, mating frequency was calculated as the ratio of TC to donor cell. At least three independent biological replicates were done.

### Cloning of *orfL* and *orfM* genes for production in *E. coli*

To produce OrfL and OrfM proteins heterologously in *E. coli*, the corresponding genes were cloned using *Nde*I and *Hind*III restriction sites into pSKB3 vector [25], in frame with a 6×His-tag and a TEV protease cleavage site. The oligonucleotides used for cloning are provided in Supplementary Table S3.

### Protein expression and purification

Overexpression and purification of recombinant RelSt3 proteins (RelSt3-FL and RelSt3_64-410_, depleted of its HTH domain) were performed as previously described in [25]. OrfL and OrfM were purified using similar conditions. Production of OrfL and OrfM in *E. coli* BL21 (*DE3*) cells was performed in LB media supplemented with kanamycin (50 μg/ml). Cells were grown at 37°C to an OD_600nm_ of ~0.5. Protein expression was then induced by the addition of IPTG (final concentration 0.5 mM) followed by incubation for 3 h at 30°C. Cells were harvested by centrifugation and stored at −80°C.

Cells overexpressing OrfL or OrfM were suspended in lysis buffer (20 ml/g of cell pellet) containing 20 mM Tris–HCl pH 7.5, 150 mM NaCl, 10 mM imidazole, 0.04 mg/ml lysozyme, 0.1% Triton X-100. The cells were lysed by sonication on ice using a Branson Sonifier, with 4 to 6 cycles of 3 × 20 s interspersed with 20-s breaks, and with an amplitude of 50%. The lysates were clarified by centrifugation for 45 min at 20 000 x *g* at 4°C. The cleared lysates were loaded on an ion metal affinity chromatography (IMAC) using a 5 ml nickel HisTrap™ HP column using an ÄKTA Prime apparatus (Cytiva) pre-equilibrated in 20 mM Tris–HCl pH 7.5, 150 mM NaCl, 10 mM imidazole. The column was washed with the same buffer containing 50 mM imidazole (10 column volumes), and then 6×His-tagged protein was eluted with a 10-column-volume gradient from 50 to 300 mM imidazole. To remove the His-tag, fractions containing OrfL or OrfM protein were cleaved by TEV protease added to a mass ratio of 1:10 (His-TEV:OrflL/M), and the mixture was dialysed against the same buffer with 10 mM imidazole for 20 h at 4°C. A reverse IMAC was next performed with a 1 ml column of Ni Sepharose^®^ (Cytiva), where the untagged OrfL/M protein does not bind anymore, whereas the His-TEV binds. Flow-through containing OrfL or OrfM was next concentrated by centrifugation using an Amicon^®^ Ultra Centrifugal Filter with 3 kDa of cut-off (Millipore) and then loaded on a HiLoad 16/600 Superdex™ 200 pg (Cytiva). The homogeneity of the eluted sample was checked by sodium dodecyl sulphate–polyacrylamide gel electrophoresis (SDS–PAGE) analysis, and the proteins were further concentrated if required.

### SEC–MALS analysis

To determine the molecular weight of the purified proteins and complexes, size exclusion chromatography coupled to multi-angle light scattering (SEC–MALS) analysis was performed. An ÄKTA Purifier™ FPLC system (Cytiva) equipped with a Superdex™ 200 Increase 10/300 GL column (Cytiva) was coupled online with miniDAWN^®^ TREOS^®^ II and Optilab^®^ T-rEX detectors (Wyatt Technology, Toulouse, France). We used 20 mM Tris–HCl (pH 7.5), 150 mM NaCl buffer with a 0.5 ml/min flow.

### Bacterial two-hybrid system

The adenylate cyclase-based bacterial two-hybrid (BACTH) technique was used to detect *in vivo* protein interactions as previously described in [38]. The genes encoding each target protein (RelSt3, OrfL, OrfM, PcrA) were cloned into vectors allowing their expression fused in N-terminal or in C-terminal to the T18 or T25 catalytic domains of the *Bordetella* adenylate cyclase. Compatible vectors producing protein fusions were transformed into the reporter DHT1 strain and the plates were incubated at 30°C for 24 h. Three independent colonies for each transformation were inoculated into 600 μl of LB medium supplemented with ampicillin, kanamycin, and IPTG (0.5 mM). After overnight growth at 30°C, 8 μl of each culture were spotted onto LB plates supplemented with ampicillin (100 μg/ml), kanamycin (50 μg/ml), IPTG (0.5 mM), and Bromo-Chloro-Indolyl-Galactopyrannoside (X-Gal, 40 μg/ml). Plates were then incubated for 16 h at 30°C. The experiments were done at least in triplicate and a representative result is shown.

### Isothermal titration calorimetry

Isothermal titration calorimetry (ITC) assays were performed using a MicroCal iTC200 (Malvern Panalytical) at 298 K with stirring at 750 rpm and reference power of 5 μcal.s^−1^. The proteins were previously dialysed against the same buffer [20 mM Tris–HCl (pH 7.5), 300 mM NaCl]. After a first injection of 0.2 µl, 39 successive injections of 1 µl were performed, with 120 s intervals between each injection. The protein nature and concentration in the shell and in the syringe are indicated in legend captions. Thermodynamic parameters ΔH (enthalpy change), n (stoichiometry), and *K*_a_ (association constant) were obtained by non-linear least-squares fitting of the experimental data using the Origin software provided with the instrument. The free energy of binding (ΔG) and entropy (ΔS) were calculated from classical equations. Experiments were carried out in triplicate and a blank titration (buffer into buffer) was subtracted.

### Binding kinetics and sizing measurements

A switchSENSE^®^ DRX instrument (Dynamic Biosensors GmbH, Munich, Germany) was used to characterise the binding kinetics of OrfL–OrfM complex and to study the OrfL–DNA and OrfM–DNA interactions [39, 40].

To study the interaction between OrfM and OrfL in real-time, OrfL was immobilised on an electro-switchable DNA chip MPC-48-2-R1-S placed into the DRX biosensor analyzer according to [40]. A 48-mer ssDNA is anchored to the chip surface and carries a fluorescent probe at its free 3′-end. A covalent conjugate between OrfL and a 48-mer complementary ssDNA was prepared with the amine coupling kit (Dynamic Bio-sensors) and purified by anion-exchange chromatography (ÄKTA-Start™ system, Cytiva). The biochip was functionalised with a conjugate concentration adjusted to 200 nM in 10 mM NaP buffer, pH 7.4, containing 40 mM NaCl, 0.05% Tween 20, 50 µM ethylenediaminetetraacetic acid (EDTA), and 50 µM EGTA (PE40 buffer). The functionalisation of the chip was verified in each experiment using sizing analysis (switchANALYSIS^®^ software). Measurements were carried out in fluorescence proximity sensing mode in real time. Serial dilutions of OrfM (from 0 to 10 µM) in PE40 buffer were injected into the microfluidics at a flow rate of 5 μl·min^−1^ for 5 min at 25°C and PE40 buffer was then injected at 30 μl·min^−1^ for 60 min. This allowed us to determine the affinity constant K_d_ of the complex from the association (k_on_) and dissociation (k_off_) kinetics constants (K_d_ = k_off_/k_on_). Data of blank controls were subtracted to normalise the signal. Potential unspecific binding of OrfM onto the DNA moiety of the nanolevers was verified with OrfL-free dsDNA nanolevers under the same experimental conditions.

The switchSENSE^®^ technology was also used to characterise the DNA–OrfL and DNA–OrfM interactions. To do so, specific oligonucleotides were designed so that they harbour a 3′-end complementary to the 5′end of the 48-mer ssDNA anchored at the surface of the biochip. We used ssDNA or dsDNA overhang oligonucleotides (Supplementary Table S3). To obtain dsDNA, 1 µM of each oligonucleotide was incubated for 30 min at room temperature in PE40 buffer. The biochip was then functionalised with 200 nM of ssDNA or dsDNA overhangs. Measurements were carried out in dynamic mode. In the presence of an electric current, DNA nanolevers oscillate at a high frequency. The binding of an analyte (here OrfL or OrfM) to the functionalised chip (here harbouring the DNA overhangs) leads to a decrease in oscillation frequency, a reduction in dynamic response, and a change in fluorescence. Different concentrations of OrfL or OrfM proteins [in 25 mM Tris–HCl (pH 8.0), 100 mM NaCl] at 25°C were used to assess the DNA-binding activity of OrfL or OrfM to the DNA overhangs. In addition, a size index for OrfL-DNA or OrfM-DNA complexes was determined using the Lollipop mathematical model [40] from fluorescence relaxation measurements of switching nanolevers recorded at interrupted flow immediately after complex association.

All curves were analysed by nonlinear fitting of single-exponential functions with the switchANALYSIS^®^ software. Each experiment was performed with two different preparations of proteins.

### NMR experiments and structure calculation for OrfM

To produce ^15^N-^13^C-labelled OrfM samples, *E. coli* BL21 (*DE3*) cells were first grown in LB preculture until they reached an OD_600_ of 0.8–1. They were then centrifuged and resuspended in M9 minimal medium supplemented with MgSO_4_, CaCl_2_, biotin, thiamine and oligoelements, and with ^15^NH_4_Cl as sole nitrogen source and ^13^C-glucose as sole carbon source. After 1 h of growth, IPTG was added to induce protein expression. Cells were harvested after an overnight culture, and OrfM was purified as described earlier, dialysed against NMR buffer (20 mM HEPES pH 7 with 100 mM NaCl), and concentrated.

NMR spectra were acquired at 298 K on a Bruker DRX 600 MHz spectrometer equipped with a TCI cryoprobe. A 0.545 mM ^15^N- ^13^C-labelled OrfM sample in NMR buffer and 10% D_2_O was used for sequential and sidechain assignment experiments, including 3D ^15^N-NOESY-HSQC, ^15^N-TOCSY-HSQC, HNHA, HNCO, HN(CA)CO, HNCA, HN(CO)CA, HNCACB, CBCA(CO)NH, HBHACONH, CC(CO)NH, H(CCO)NH, and 3D ^13^C-NOESY-HSQC. Backbone dynamics were analysed using ^15^N longitudinal relaxation rate *R*_1_ (relaxation delays of 2, 10, 50, 100 (x2), 200, 400, 600, 1000, and 1400 ms), ^15^N transverse relaxation rate *R*_2_ (relaxation delays of 0, 17 (x2), 34, 51, 68, 85, 102, 119, and 136 ms), and ^1^H-^15^N heteronuclear NOE experiments. Measurements were recorded on a 0.15 mM ^15^N-labelled sample in phosphate buffer (pH 6.5) with 200 mM NaCl and 10% D_2_O. Spectra were processed using Topspin 3.0 software (Bruker) and analysed with NmrViewJ [41] and CcpNmr [42].

679 distance restraints derived from 3D ^15^N-NOESY-HSQC or 3D ^13^C-NOESY-HSQC spectra, 74 φ/Ψ dihedral angles generated by DANGLE [43], and 25 hydrogen bonds were input as restraints in ARIAweb server [44, 45]. Among the 100 structures generated by ARIA2, 10 models of lowest energy were refined in water. OrfM global correlation time was determined using TENSOR2 [46], excluding the residues with HetNOE < 0.6 or *R*_2_/*R*_1_ ratio more than one standard deviation away from the mean value.

OrfM-assigned chemical shifts are deposited in the Biological Magnetic Resonance Data Bank database (accession code 34970) and structural coordinates in the Protein Data Bank (accession code 9HNJ).

### Protein structure predictions

Structure predictions were performed using AlphaFold3 [47]. Various statistical AlphaFold scores have been used to evaluate structure predictions: pLDDT is a per-atom score (0–100) of a predicted model, where a high value indicates great confidence; pTM (0–1) is a global score for the predicted fold, and a pTM value above 0.5 indicates a likely correct overall fold; ipTM is a score (0–1) that estimates the confidence in the quaternary structure of proteins. According to the recommendations of the AlphaFold authors, values above 0.8 represent high-quality predictions, while values below 0.6 are likely to indicate a failed prediction.

### Hydrodynamic calculations

Global correlation times τ_c_ were calculated from the rotational diffusion coefficients *D* determined at 298 K by HYDRONMR [48] using the equation τ_c_ = (6*D*)^−1^, as in TENSOR2 [46]. For the monomeric state, τ_c_ average value and standard deviation were calculated using the 10 NMR structural models of OrfM. For the dimeric state, an AlphaFold model of a homodimeric OrfM was used.

### Electrophoretic mobility shift assays

Electrophoretic mobility shift assays (EMSA) were performed in 5% native acrylamide gels. The DNA substrate was an oligonucleotide labelled at its 5′ end with 6-FAM (Eurogentec, France), except for the complementary strand (in case of dsDNA) that was unlabelled (Supplementary Table S3). Reactions were performed in 20 µl final volume containing 0.2 µM of oligonucleotides (ssDNA or dsDNA) in the presence of increasing concentrations of the respective proteins (OrfL, OrfM, or the OrfL–OrfM complex; see figure legends). Reaction buffer contained 20 mM HEPES and 150 mM NaCl. The mixtures were incubated for 15 min at 37°C. Next, 0.02% paraformaldehyde was added to the mix and a second incubation of 1 h at 37°C was performed. Two microlitres of blocking solution (1 M Tris–HCl pH 6.8) was then added, followed by an additional incubation for 10 min at room temperature. 2.5 μl of loading buffer (2.5 mg/ml bromophenol blue and 0.4 g/ml sucrose) were then added. Prior to sample loading, gels were pre-run in miniPROTEAN (Bio-Rad) cells at 4°C for 1 h at 84 V in 0.5X Tris–borate–EDTA buffer. Samples were then run at 4°C for 1 h 30 min at 84 V. Labelled DNA was visualised using the ChemiDoc XRS system (Bio-Rad) and images were analysed using the Image Lab software (Bio-Rad). At least three replicates were performed for each experiment.

### Supercoiled DNA relaxation assays

Supercoiled plasmidic DNA (200 ng) of pBR322-*oriT* [25] and 1.6 μM of RelSt3 were incubated for 20 min at 30°C with increasing amounts of OrfL, OrfM, or the OrfL–OrfM complex in a reaction buffer containing 20 mM Tris–HCl (pH 7.5), 200 mM NaCl, 5 mM MnCl_2_, 0.05 mM EDTA, 1% glycerol (w/v) and 1 mM β-mercaptoethanol, with a final volume of 20 μl. Reactions were stopped by addition of final concentrations of 0.5% SDS and 100 μg/ml of proteinase K, followed by a second incubation at 30°C for 20 min. The products of the reaction were electrophoresed on 1% agarose gel in 1× Tris-Borate-EDTA for 20 h at 2.5 V/cm and the gel was stained with ethidium bromide. Gel pictures were captured using a Uvidoc HD6 system (Uvitec, Cambridge).

### Oligonucleotide cleavage and strand transfer assays

Oligonucleotide cleavage and strand transfer assays were performed as previously described [27]. The reaction mixtures contained the indicated oligonucleotides (2 pmol each), 3.2 µM of RelSt3 and increasing concentrations of the respective proteins (OrfL, OrfM or the OrfL–OrfM complex; see figure legends) in 18 µl final volume. Reaction buffer contained 20 mM Tris–HCl, 150 mM NaCl, and 5 mM MnCl_2_. Mixtures were incubated for 30 min at 37°C and treated with proteinase K (1 mg/ml, final concentration) for a second 15 min incubation at 37°C. Two microlitres of loading buffer (2.5 mg/ml bromophenol blue and 0.4 g/ml sucrose) were then added. Prior to loading samples, 5% acrylamide gels were pre-run as described for EMSAs, and samples were then run for 1 h 30 min at 84 V. Labelled DNA was visualised using the ChemiDoc XRS system (Bio-Rad) and band intensity was quantified with the Image Lab software (Bio-Rad). At least three replicates were performed for each experiment.

## Results

### Both OrfL and OrfM proteins are essential for ICE*St3* conjugation

To assess the significance of OrfL and OrfM proteins in the conjugation process of ICE*St3*, we generated two mutant strains of *S. thermophilus* LMG18311 harbouring ICE*St3* and in which either the *orfL* gene or the *orfM* gene was deleted. Both mutants were designed to prevent any polar effect on downstream genes during the expression of the operon harbouring the conjugative genes of ICE*St3*. Mating experiments were carried out using as donor strains either the *S. thermophilus* LMG18311 strain harbouring the wild-type ICE*St3* or strains harbouring a mutated ICE*St3* version (Δ*orfL* or Δ*orfM*). The conjugative transfer frequency of wild-type ICE*St3* was 1.7 × 10^−5^ ± 2.1 × 10^−6^ transconjugants per donor cell. In contrast, the transfer of the ICE*St3*Δ*orfL* mutant and of the ICE*St3*Δ*orfM* mutant was undetectable (with detection thresholds ≤ 5.0 × 10^−8^ transconjugants per donor cell). We constructed strains complemented with the deleted gene (*OrfL* or *OrfM*), and for both of them the conjugation frequency was restored to the level observed with the wild-type ICE*St3*, with transfer frequencies of 1.1 × 10^−5^ ± 9.2 × 10^−7^ and 1.0 × 10^−5^ ± 6.7 × 10^−7^ transconjugants per donor cell, respectively. Based on these results, we concluded that both OrfL and OrfM are strictly required for the conjugative transfer of ICE*St3*.

### OrfM and OrfL are mainly monomeric in solution

**Figure 1.**
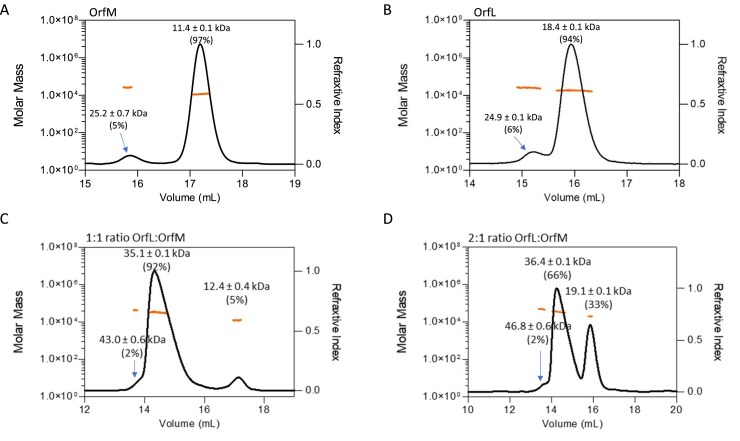
Analysis of oligomerisation of isolated and mixed OrfM and OrfL proteins by SEC–MALS. SEC MALS elution analyses are shown for (**A**) OrfM at 30 µM, (**B**) OrfL at 30 µM, (**C**) 1:1 OrfL:OrfM molar ratio, and (**D**) 2:1 OrfL–OrfM molar ratio. Molecular weight was calculated by MALS (red lines) and was plotted on a logarithmic scale (Da, left-hand *y*-axis) against elution time (*x*-axis). The refractive index is shown on the right-hand *y*-axis. Molecular weight and contribution in mass fraction (in %) are indicated at the top of each corresponding peak. In panels (A) and (B), the insets correspond to SDS–PAGE analysis of the fraction corresponding to the main peak.

To better understand the role of OrfL and OrfM proteins in the conjugation process, we were first interested in characterising their oligomeric state in solution. For that purpose, OrfL and OrfM were overexpressed and purified from *E. coli* as described in the ‘Materials and methods’ section. According to our analysis using SEC–MALS technology, OrfM protein (theoretical MW of 11.0 kDa) was present in solution mainly as a monomer (Fig. 1A, 11.4 kDa), with a limited population corresponding to a dimer (~5% of the protein injected, 25.2 kDa). The purified OrfL protein (theoretical MW of 17.5 kDa) was also found to be mainly monomeric in solution with a limited proportion corresponding to a dimer (Fig. 1B). These results indicated that OrfM and OrfL are both mainly monomeric in solution, but are also able to form a limited proportion of dimers under our conditions.

### OrfL and OrfM form a complex

**Figure 2.**
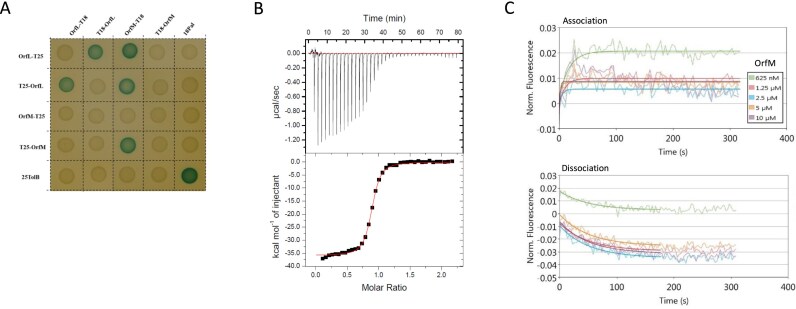
OrfL and OrfM form a complex *in vivo* and *in vitro*. (**A**) Bacterial two-hybrid assays. Reporter *E. coli* cells producing the indicated proteins fused to the T18 or T25 domain of the adenylate cyclase were spotted on LB plates complemented with X-gal, where blue colour indicates the interaction between the two proteins. (**B**) Characterisation of the thermodynamic parameters of the OrfL–OrfM interaction using ITC. Binding isotherms and thermograms at 298 K were obtained by injecting OrfM at 280 µM into the shell containing OrfL at 28 µM. (**C**) Characterisation of the kinetics parameters of the OrfL–OrfM interaction in real time using switchSENSE technology. The biochip was functionalised with OrfL as described in the ‘Materials and methods’ section. Association (upper panel) was analysed by injecting OrfM in the microfluidics at different concentrations (from 0.625 to 10 µM; see inset for legend). Dissociation (bottom panel) was monitored following each association. The signal is expressed as normalised fluorescence change.

We investigated the putative interaction between OrfL and OrfM by *in vivo* and *in vitro* approaches. We first used bacterial two-hybrid approach (BACTH), an *in vivo* method that tests protein–protein interactions in *E. coli*. OrfL and OrfM were fused to the N- or C-terminus of the *Bordetella pertussis* Cya adenylate cyclase T25 or T18 domains. To monitor positive interaction, *E. coli* clones harbouring compatible fusion proteins were grown on X-Gal-containing reporter plates, and blue-coloured colonies were sought (Fig. 2A). A clear self-interaction was identified for OrfM and for OrfL, confirming the SEC–MALS data reporting their multimerisation. Interestingly, BACTH assays also revealed a cross-interaction between OrfM and OrfL (Fig. 2A).

We next attempted to validate the interaction between the purified OrfL and OrfM proteins by co-purification. OrfL and OrfM were mixed in a 1:2 molar ratio, and the mixture was submitted to SEC analysis. We obtained two major peaks (Supplementary Fig. S1). Subsequent SDS–PAGE analysis revealed that both OrfL and OrfM were present in the first peak, possibly corresponding to the OrfL–OrfM complex. Instead, only OrfM was detected in the second peak, suggesting an excess of OrfM relative to OrfL in this experiment.

The OrfL–OrfM complex assembly was next examined by SEC–MALS. As depicted in Fig. 1 CD, two different molar ratios were tested to estimate the interaction stoichiometry: 1:1 and 2:1 OrfL:OrfM. With both ratios, a complex of ~35 kDa was observed (accounting for 92% of the sample for the 1:1 ratio), which could correspond to a 1:1 OrfL:OrfM heterodimer. At both ratios, the peak corresponding to the heterodimer stretches on the right, suggesting unspecific interactions of the complex with the chromatography matrix and/or partial dissociation of the complex during SEC migration. Interestingly, under the 1:1 ratio condition, almost no excess of individual OrfL or OrfM protein was detected (Fig. 1C), in agreement with a 1:1 stoichiometry for the OrfL–OrfM complex. Indeed, only 5% of the sample corresponded to a 12.4 kDa species, fitting with the OrfM monomer. In contrast, under the 2:1 OrfL:OrfM ratio condition (Fig. 1D), all OrfM species were incorporated into the OrfL–OrfM complex, while a peak at 19.1 kDa was observed, probably corresponding to the OrfL monomer (33%). This suggests that OrfL was largely over-represented in this 2:1 ratio condition, further supporting a 1:1 molar ratio for the OrfL–OrfM complex. In both ratio conditions, a minor peak with a higher molecular weight of 43.0 kDa or 46.8 kDa was detected. This peak may represent an OrfM dimer in complex with an OrfL monomer (2 × 12.4 + 19.2 kDa), suggesting that OrfM in its dimeric form could also interact with OrfL at high concentrations. Altogether, these *in vitro* results support the formation of an OrfL–OrfM complex, corroborating the findings from the BACTH approach.

The interaction between OrfL and OrfM was next assessed by ITC, which is a method of choice to study the interaction of purified proteins, providing a detailed thermodynamic characterisation. Fig. 2B presents the binding reaction (top panel) and the thermogram (bottom panel) obtained from the interaction between OrfL and OrfM. Data analysis using the NITPIC software [49] suggested that the interaction fits better with a single binding site model. The estimated association constant K_a_ was 7.1 ± 0.4 × 10^6^ M^−1^ (K_d_ ≈ 140 nM). The estimated stoichiometry was *n* = 0.87, consistent with a 1:1 OrfL–OrfM complex, as deduced from the SEC–MALS analysis. The deduced thermodynamic parameters are provided in Table 1. The substantial negative enthalpy suggests a high number of hydrogen bonds and van der Waals forces involved in the complex formation. The negative entropy of the reaction (unfavourable) suggests a low hydrophobic contribution and/or major conformational changes, possibly leading to reduced flexibility upon complex formation.

**Table 1.** Thermodynamic parameters of the interaction between OrfL and OrfM as deduced from ITC data

Parameter	Ka (M^−1^)	ΔH (kcal/mol)	−TΔS (kcal/mol)	ΔG (kcal/mol)	n
OrfL–OrfM	7.1 × 10^6^ ± 4.6 × 10^5^	−35.9 ± 0.016	26.6	−9.3	0.87

Finally, to gain further insights into the kinetics of the complex formation, we analysed the OrfL–OrfM interaction in real time using the switchSENSE technology [40]. OrfL was covalently attached to the DNA nanolever cNLB48, as described in the ‘Materials and methods’ section, and OrfM was introduced into the flow at various concentrations. The constants determined from the sensorgrams (Fig. 2C) indicated a rapid association of OrfM (*k*_on_ = 6.2 ± 1.2 × 10^4^ M^−1^ s^−1^) and a rapid dissociation when OrfM was replaced by the buffer in the microfluidics (*k*_off_ = 1.9 ± 0.1 × 10^−2^ s^−1^). The resulting *K*_d_ was estimated to be 311 ± 62 nM, in the same order of magnitude as the value determined using ITC, which corroborates a strong interaction between OrfL and OrfM proteins.

### OrfM and OrfL belong to OB-fold proteins

**Figure 3.**
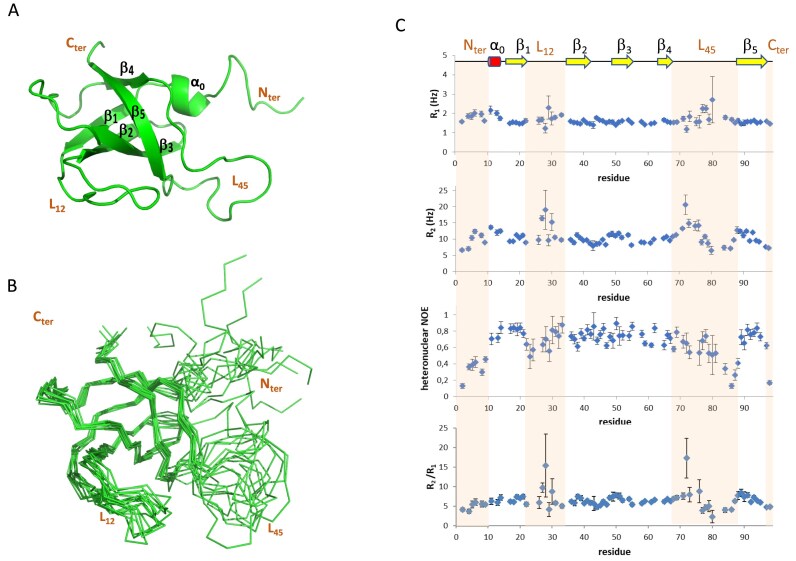
OrfM NMR structure and dynamics. (**A**) Medoid conformer showing an OB-fold α_0_-β_1_-β_2_-β_3_-β_4_-β_5_. (**B**) NMR ensemble of 10 conformers. (**C**) ^15^N *R*_1_ and *R*_2_ relaxation rates, ^1^H-^15^N heteronuclear NOE (HetNOE), and *R*_2_/*R*_1_ ratios for OrfM at a protein concentration of 0.15 mM in Tris–HCl (pH 7.5) with 200 mM NaCl at 298 K. HetNOE values below 0.6, corresponding to flexible regions, are observed at the N- and C-termini (residues 2–9 and 98), in L_45_ (residues 73–76, 79–88), and in L_12_ (residues 23–24 and 29). Residues in L_12_ (residues 27, 28, and 30) and L_45_ (residues 72 and 76) exhibit *R*_2_/*R*_1_ ratios above the average by more than one standard deviation, indicating the contribution of a chemical exchange term in *R*_2_ value, which is usually attributed to slow conformational exchange at the μs-ms time-scale [86].

To further investigate the function of OrfM, we initially attempted to determine its 3D structure by X-ray crystallography. However, due to unsuccessful crystallisation trials, we shifted to NMR spectroscopy as an alternative approach. Standard 2D and 3D NMR experiments were carried out on a ^15^N- and ^13^C-labelled sample, and the resulting data were analysed to determine the structure of OrfM (Fig. 3A). Backbone assignment was obtained for all residues except for T56, S74, and N79. Superposition of the NMR ensemble of models reveals several ill-defined regions with high RMSD: the first 14 N-terminal residues, the C-terminal residues (97–98), and two loops (residues 23–32 and 73–86) referred to as L_12_ and L_45_ (Fig. 3B). Few NMR restraints could be obtained for these regions (see Supplementary Table S4 for NMR structure statistics), especially for a dozen of residues located in these loops, which gave extremely broad and weak signals, as seen in the ^15^N-HSQC spectrum (Supplementary Fig. S2). ^15^N relaxation measurements enabled the identification of poorly or unstructured residues in these four regions and the detection of segments in slow conformational exchange in both L_12_ and L_45_ loops (Fig. 3C). Besides, an overall correlation time of 7.4 ± 0.1 ns at 298K could be inferred from relaxation data using TENSOR2 [46]. This value is in agreement with that determined by HYDRONMR [48] for OrfM in a monomeric state (8.2 ± 0.7 ns), whereas that expected for a dimer was estimated at 14 ns. These results therefore indicate that OrfM is mainly monomeric in the experimental conditions used for this NMR study.

The solution structure of OrfM calculated from NMR-derived constraints was used to search for closely related structural homologs in the PDB using the DALI server [50]. The first 10 hits had close *z*-score values, ranging from 6.3 to 6.8, and all corresponded to oligosaccharide/oligonucleotide-binding (OB) fold domains of proteins involved in DNA or RNA processing, such as archaeal Replication Protein A (RPA, PDB 3E0E or 8AAS) [51], bacterial SSBs (4DAM) [52], or the bacterial editosome (4DK6) [53]. Indeed, the typical β-barrel of OB-folds composed of five beta strands arranged in an overall 1–2–3–5–4–1 topology [33, 34] was found in the OrfM structure (Fig. 3A), confirming its classification as an OB-fold protein. However, several peculiarities can be highlighted in OrfM structure compared to the canonical OB-folds: (i) an additional helix turn α_0_ (residues 10–13) is located just before β_1_, while it is usually absent in OB-fold proteins; (ii) a long loop between β_1_ and β_2_ (referred to as L_12_) containing a β-hairpin-like structure tilted relative to the β_1_-β_2_ sheet; (iii) the α-helix between β_3_ and β_4_ that usually caps the barrel in canonical OB-fold domains is absent.

A 3D structural model was predicted for OrfM using AlphaFold3 (Supplementary Fig. S3). It exhibited high accuracy with a pTM score of 0.8, and it was consistent with the NMR structure. The comparison between the AlphaFold model and the NMR structure revealed that the only difference is located at the N-terminal segment of OrfM.

Interestingly, among the top 10 hits found using DALI, the NMR structure of SAG0934 protein from *S. agalactiae* (PDB 2K5D) stood out from the others, with a relatively high percentage identity with OrfM (26% against other values below 15%). This protein structure can be assimilated to Orf22 of Tn*916* from *Enterococcus faecalis* [18], differing only by the absence of the 20 C-terminal residues. Orf22 has no additional secondary structure element in its disordered N-terminal region. Its β_1_-β_2_ sheet is slightly longer and can be considered as a single relatively straight sheet, in contrast to that of OrfM, where it significantly bends towards the β-barrel (Supplementary Fig. S3). No dynamics NMR data are available for Orf22; however, the very good superposition of the NMR models suggests no significant mobility in the β1-β2 sheet. In Orf22 structure, the loop L_45_ contains an additional helical turn, and it is less disordered than in OrfM. It is important to note that Tn*916* encodes two OB-fold proteins, as ICE*St3* does. They are encoded by genes arranged in a conserved synteny (Supplementary Fig. S4). Based on size and position of their encoding genes, OrfM would be more appropriately compared to Orf23, and OrfL to Orf22. Unfortunately, no experimental 3D data are currently available for Orf23 nor for OrfL proteins. A multiple sequence alignment was performed to assess the relationship between these proteins. They exhibit low sequence conservation in terms of primary amino acid sequence (Supplementary Fig. S4). Interestingly, OrfL and OrfM were more distant from their respective counterparts from Tn*916* or ICE*Bs1*, and even from each other (with only 19.6% of amino acid sequence identity, Supplementary Fig. S4C). As Orf22 and Orf23 sequences are fairly close homologues to each other compared with OrfL (which is more distantly related), the structural comparison between OrfM and Orf22 NMR structures remains relevant.

NMR experiments were also performed to investigate the interaction of OrfM with OrfL. However, the addition of OrfL caused extreme broadening of OrfM signals in the ^15^N-HSQC spectrum without any observable chemical shift variation or reappearance of peaks corresponding to the bound state. This behaviour is consistent with the formation of a complex at an intermediate exchange rate [54], which aligns with the k_on_ and k_off_ values obtained by switchSENSE. Unfortunately, this phenomenon hindered the mapping of OrfM interaction region with OrfL by NMR. A 3D structural model was first predicted for OrfL using AlphaFold3 (pTM = 0.66), which turned out to harbour an OB-fold domain (Supplementary Fig. S3). We then generated an AlphaFold 3 model of OrfL–OrfM complex. In this model obtained with a highly satisfactory score (see Supplementary Table S5), a pseudo-2-fold axis connects the two proteins, such that their N-termini (including α_0_ helix turns of both proteins) and their loops L_45_ form contacts in an antiparallel arrangement. This arrangement is similar to that observed in X-ray structures of dimeric or tetrameric OB-fold proteins (Supplementary Fig. S5E and F). Homodimers and tetramers of OrfM, OrfL, and their homologs (i.e. Orf22 and Orf23 from Tn*916* and HelP from ICE*Bs1*) were predicted as well (Supplementary Table S5). Medium to high scores were obtained for Orf22–Orf23, Orf23–Orf23, and HelP–HelP dimers. These AlphaFold models suggest a similar association, which would, however, need to be confirmed experimentally.

### OrfL and OrfM bind DNA weakly and without sequence specificity

**Figure 4.**
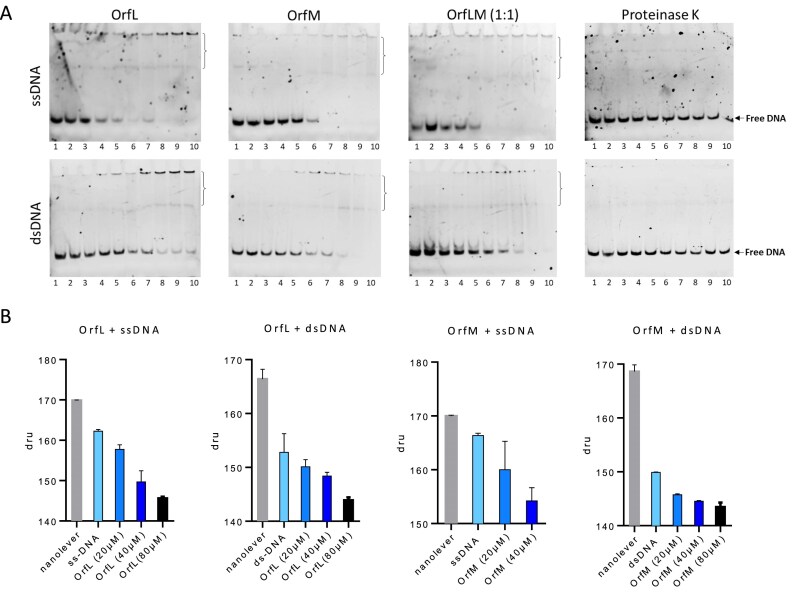
OrfL and OrfM bind DNA weakly. (**A**) EMSA performed with OrfL or OrfM protein pre-incubated with DNA (0.2 µM) in the presence of 0.02% of paraformaldehyde. Increasing concentrations of protein were applied as follows: lane 1, 0 µM; lane 2, 0.2 µM; lane 3, 0.6 µM; lane 4, 1.2 µM; lane 5, 2 µM; lane 6, 4 µM; lane 7, 6 µM; lane 8, 8 µM; lane 9, 10 µM; lane 10, 12 µM. (**B**) The results are represented by plotting the control conditions (nanolever or overhangs DNA free of analytes) and the used analyte (OrfL or OrfM at increasing concentrations) on the *x*-axis and the dynamic response on the *y*-axis (dru: dynamic response unit). The DNA type (ss or ds) and the protein used are indicated at the top of each batch of experiments. The error bar corresponds to the standard deviation obtained for 3 experiments (note that 7 measures are performed for each experiment).

Given that many OB-fold proteins have been described as ssDNA and/or dsDNA binding proteins [33, 55, 56], we decided to investigate if OrfL and/or OrfM proteins could bind to DNA. In our initial EMSA assays using various ssDNA or dsDNA substrates, we did not observe any notable DNA binding, either with the isolated proteins or with the pre-formed OrfL–OrfM complex (data not shown). However, the addition of very low concentrations of para-formaldehyde (0.02% PFA) enabled band shift for both ssDNA and dsDNA to be visualised in the presence of OrfL, OrfM, or the pre-formed OrfL–OrfM complex (1:1 ratio) (Fig. 4A). Since such cross-linking agents are commonly used to reveal and stabilise interactions between different biomolecules and also for DNA–protein interactions [57–59], these results suggest that interactions between OrfL/OrfM and DNA are probably weak under the experimental conditions used in our assay. However, each of the proteins, as well as the complex, appeared to display slightly higher affinity for ssDNA. These results correlate with the known behaviour of proteins harbouring an OB-fold motif towards ssDNA [60].

To confirm these interactions with DNA using an alternative approach, we again used the switchSENSE, taking advantage of the fact that this technology uses DNA as bait. After functionalisation of the biochip by ssDNA or dsDNA overhangs, assays were performed with increasing concentrations of OrfL or OrfM (Fig. 4B). We observed a decrease in dynamic response upon addition of OrfL or OrfM proteins, compared to DNA overhangs alone. These results indicate that OrfL and OrfM could interact with both dsDNA and ssDNA. We observed similar patterns with different DNA sequences, which suggests that these bindings are not sequence specific. At a protein concentration of 80 µM, the sizing index of the dsDNA–protein complex was calculated. The sizing indexes obtained were 6.95 ± 0.07 and 6.55 ± 0.35 for dsDNA-OrfM and dsDNA-OrfL complexes, respectively. In contrast, it was 5.95 ± 0.21 with dsDNA alone (without protein). We concluded that the sizing index increased with the addition of OrfL or OrfM, which thus confirms the formation of a DNA–protein complex on the DNA overhang.

### OrfL interacts with RelSt3 and PcrA

**Figure 5.**
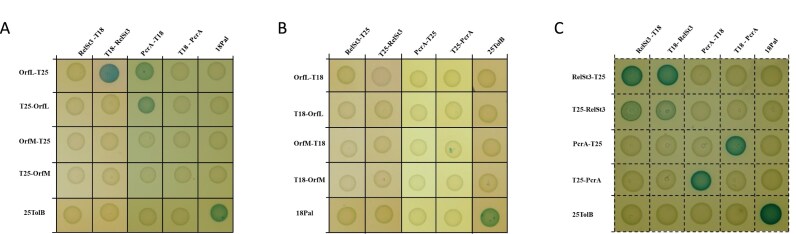
OrfL interacts with RelSt3 and PcrA. BACTH analysis was performed to test the interactions between (**A** and **B**) OrfL or OrfM proteins and RelSt3 or PcrA proteins, and (**C**) between RelSt3 and PcrA proteins. *E. coli* clones expressing the corresponding proteins were spotted on the same plates as described in Fig. 2A: (A–C) LB plates complemented with X-gal.

We next investigated potential interactions of OrfL/OrfM proteins with other relaxosome candidate partners involved in ICE*St3* DNA processing. BACTH analysis revealed that OrfL could interact with RelSt3 and with PcrA, an SFI helicase encoded by *S. thermophilus* genome (Fig. 5A). In contrast, OrfM did not show any interaction with either RelSt3 or PcrA (Fig. 5A and B). We seized the opportunity to have on hand the required constructs to test a putative interaction between RelSt3 and PcrA. This RelSt3–PcrA interaction was not detected by our BACTH results (Fig. 5C). Instead, we confirmed by this approach the ability of both RelSt3 and PcrA to self-oligomerise (Fig. 5C). Indeed, we previously demonstrated that RelSt3 forms a dimer in solution [25]. In turn, PcrA was previously proposed to form dimers as an active form [61].

Although the BACTH assay indicates possible interactions between OrfL and the two other proteins, RelSt3 or PcrA, these could not be confirmed by *in vitro* approaches. This experimental discrepancy could be attributed to specific properties of RelSt3: (i) its requirement for high salinity (~500 mM NaCl) to maintain stability and (ii) its propensity to precipitate upon stirring, rendering standard approaches such as microcalorimetry, SEC, or pull-down assays impractical. To circumvent this problem, a truncated version of RelSt3 (RelSt3_64-410_, depleted of its N-terminal HTH domain) [27] with slightly improved stability (although still requiring 300 mM NaCl) was used in ITC assays, but no interaction could be detected with OrfL, OrfM, or the OrfL–OrfM complex (Supplementary Fig. S6). Once again, the fairly high salinity condition used could be the reason why no interactions were observed, and we do not rule out possible weak and/or labile interactions. Besides, we were unable to purify sufficient quantities of *S. thermophilus* PcrA from *E. coli* for *in vitro* interaction assays.

### OrfL and OrfM stimulate the single-stranded endonuclease activity of RelSt3

**Figure 6.**
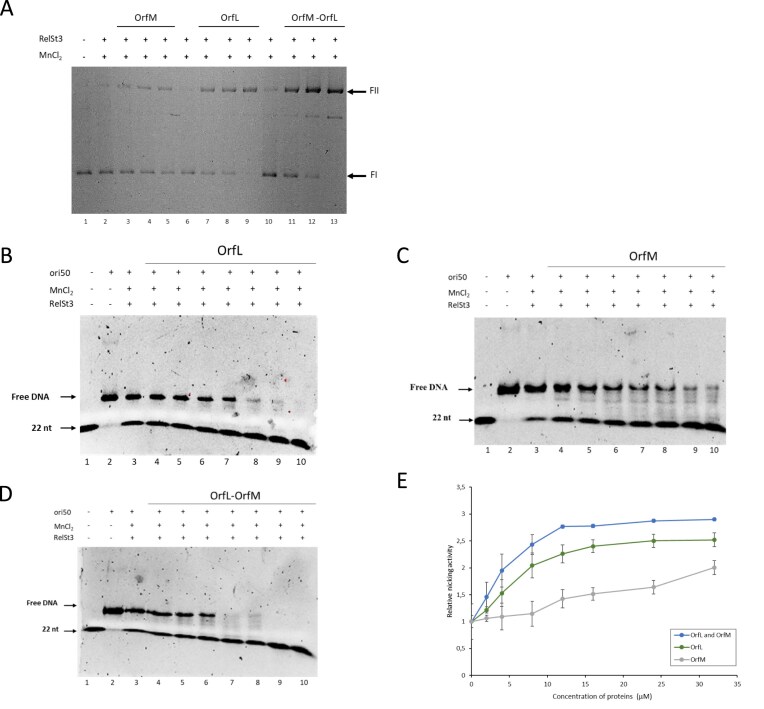
OrfL and OrfM stimulate the single-stranded endonuclease activity of RelSt3. (**A**) Plasmid relaxation assays in the presence of OrfL, OrfM, or the pre-formed complex OrfL–OrfM. The same concentration of RelSt3 was used in each sample. Lane 1: plasmid alone (no protein). Lanes 2, 6, and 10: RelSt3 alone (controls). Four micromolar of OrfM, OrfL, or OrfL–OrfM were, respectively, added in lanes 3, 7, and 11. Eight micromolar of OrfM, OrfL, or OrfL–OrfM were, respectively, added in lanes 4, 8, and 12. Sixteen micromolar of OrfM, OrfL, or OrfL–OrfM were, respectively, added in lanes 5, 9, and 13. FI stands for plasmidic form I (supercoiled), and FII stands for plasmidic form II (nicked). (**B**) Nicking assays performed with the ori50 substrate as described previously [27]. Lane 1: labelled 22 nt ssDNA alone as marker. Lane 2: ori50 substrate alone. Lanes 3 to 10: the same concentration of RelSt3 was used (1.6 µM). Increasing concentrations of OrfL were added as follows: lane 3, 0 µM; lane 4, 2 µM; lane 5, 4 µM; lane 6, 8 µM; lane 7, 12 µM; lane 8, 16 µM; lane 9, 24 µM; lane 10, 32 µM. (**C**) Same as (B) with OrfM as auxiliary protein. (**D**) Same as (B) with OrfL–OrfM complex as auxiliary protein. (**E**) Graph representing the nicking activity of RelSt3 against the concentration of the respective auxiliary protein (OrfL, OrfM, or OrL–OrfM complex). The nicking activity was normalised with the activity observed for RelSt3 without auxiliary protein. The standard deviation for 3 experiments is shown.

As OrfL could interact with RelSt3, we hypothesised that the OrfL–OrfM complex may regulate DNA processing during conjugation by modulating the biochemical activity of RelSt3, as it has been previously demonstrated for various relaxosomal auxiliary proteins. We first tested whether OrfL/OrfM could modulate the nicking activity of RelSt3 using as a substrate a supercoiled plasmidic DNA harbouring the *oriT* sequence of ICE*St3* [25]. An enhanced nicking activity of RelSt3 was observed in the presence of increasing concentrations of OrfM (Fig. 6A). We observed a higher stimulation using increasing concentrations of OrfL, and a slight cumulative effect was observed when using increasing concentrations of the OrfL–OrfM complex. To confirm these effects, we performed single-stranded endonuclease assays with our chimeric ori50 substrate. As described in [27], this substrate harbours, on the right-hand side, the double-stranded *bind* site of RelSt3, ensuring a correct fixation of the RelSt3 HTH domain, and on the left-hand side, the *nic* region as ssDNA, allowing easy visualisation of the cleavage with a 5′ fluorescent labelling (6-FAM). RelSt3 cleavage of ori50 substrate generates a short labelled 22 nt ssDNA fragment, which migrates more rapidly on acrylamide gel [27]. As observed for relaxation experiments, a higher stimulation of the single-stranded endonuclease activity was found for OrfL compared to OrfM (Fig. 6BC), with a slight cumulative effect when OrfL was in complex with OrfM (Fig. 6DE). No stimulation of RelSt3 nicking activity was observed when BSA (bovine serum albumin) or the OrfG protein (the VirB8-like protein encoded by ICE*St3*) [62] were used as control proteins (Supplementary Fig. S7). These results demonstrate that OrfL, OrfM, and OrfL–OrfM complex stimulate the production by RelSt3 of the specific nick on *oriT* in a concentration-dependent manner.

### OrfL and OrfM reduce the strand transfer activity of RelSt3

**Figure 7.**
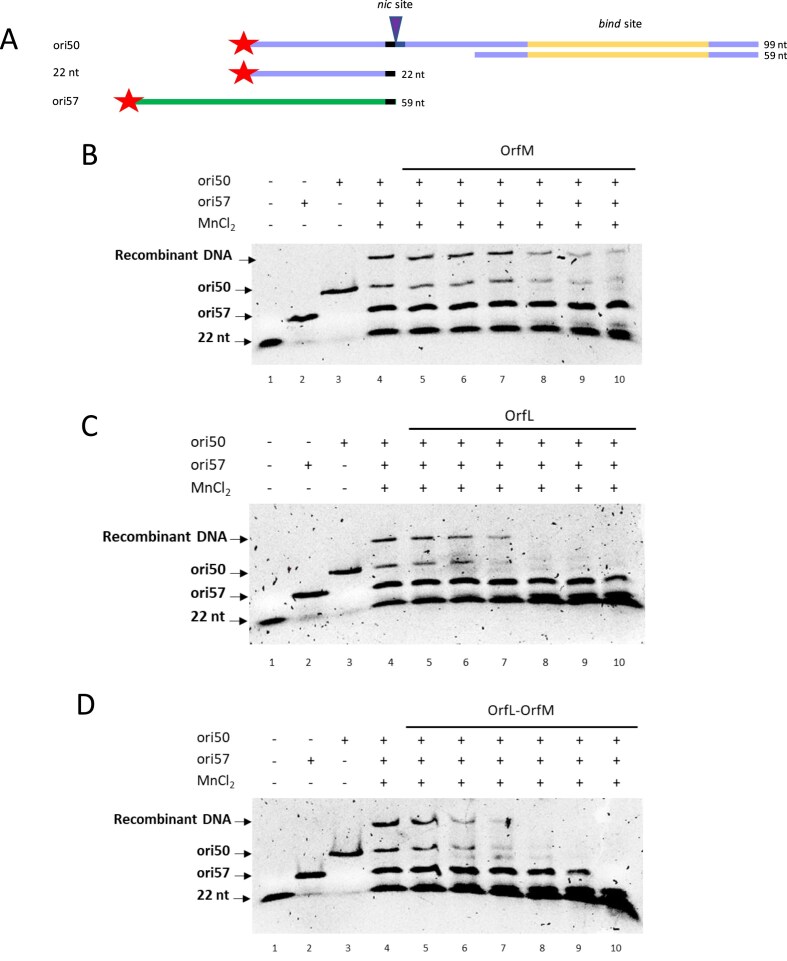
OrfL and OrfM reduce the strand transfer activity of RelSt3. (**A**) Organisation of ori50 and ori57 as described previously [27]. (**B**) Nicking-closing assays performed in the presence of RelSt3 (3.2 µM), ori50, ori57, and with increasing amounts of OrfM protein. Lane 1, 22 nt ssDNA marker; lane 2, 59 nt ssDNA ori57; lane 3, ori50. Lanes 4–10: the same concentration of RelSt3 was used in the presence of ori50 and ori57. Increasing concentrations of OrfL were added as follows: lane 4, 0 µM; lane 5, 2 µM; lane 6, 4 µM; lane 7, 8 µM; lane 8, 12 µM; lane 9, 16 µM; lane 10, 24 µM. (**C**) Same as (B) with OrfM as auxiliary protein. (**D**) Same as (B) with OrfL–OrfM complex as auxiliary protein.

We previously demonstrated that RelSt3 was able to catalyse a strand transfer reaction [27], which reflects the recircularisation of the incoming ssDNA in the recipient cell. This strand transfer activity can be evidenced by the detection of recombinant DNA in the presence of a longer oligonucleotide labelled at the 5′-end and harbouring half of the *nic* site at its 3′-end (ori57, Fig. 7A). The addition of increasing concentrations of OrfM protein slightly reduced the strand transfer activity of RelSt3 (Fig. 7B), while increasing concentrations of OrfL or of the OrfL–OrfM complex displayed stronger yet comparable reducing effects (Fig. 7C and D). This observed inhibition of strand transfer activity could, at least partially, explain the apparent stimulation of the RelSt3 nicking activity. Indeed, if more DNA molecules cannot be religated in the presence of OrfL/OrfM, the equilibrium shifts towards the accumulation of cleaved DNA.

## Discussion

The OB-fold was first identified 30 years ago [63], and it turned out to be found in a variety of proteins. This versatile structural domain is present, to name a few examples, in anticodon-binding domains of aminoacyl-tRNA synthetases, bacterial superantigen toxins and bacterial AB_5_ toxins, inorganic pyrophosphatases, and various proteins interacting with DNA [32]. DNA-interacting proteins containing OB-folds are often referred to as genome guardians due to their crucial roles in central DNA metabolism, including replication, recombination, and repair [33, 34]. These genome guardian proteins harbour one or several OB-fold domains, and they are found in all living cells across the three domains of life [33, 34, 55]. Notable examples include the eukaryotic BRCA2 protein involved in various cancers, the DNA ligase 4, the archaeao-eukaryotic MCM replicative helicase, the bacterial DNA polymerase II, and the bacterial DNA repair RuvA protein [34]. Another significant set of genome guardian proteins are the single-stranded DNA binding proteins (SSB). These are found in Eukarya and Archaea as the RPA [64, 65], in Bacteria as the SSB proteins [66], and also in viruses, as the gp32 from the bacteriophage T4 [67]. It is important to note that in many cases, the OB-fold domain is not restricted to ssDNA binding, but can also be involved in dsDNA binding and/or in protein interactions [34, 55].

In bacterial conjugation, numerous plasmids from Gram-negative bacteria encode an SSB protein, including the prototypical F plasmid from *E. coli* [68]. Interestingly, the gene encoding this SSB protein is located in the leading region of the plasmid, which first enters the recipient cell during conjugation. This positioning allows for immediate transcription upon entry while the DNA is still single-stranded [69, 70]. This SSB protein is thought to protect ssDNA during conjugation in both donor and recipient cells, and to suppress the SOS response induced by mating in the recipient cell [71, 72]. Recently, another OB-fold protein has been described in Gram-positive bacterial conjugation, the protein PrgE of the pCF10 plasmid from *Enterococcus faecalis* [60]. Surprisingly, despite displaying weak sequence similarities with SSB encoded by *E. faecalis* genome, PrgE turned out to bind both ssDNA and dsDNA equally well. The 3D crystal structure of PrgE bound to ssDNA depicted a singular binding, forming a string of pearls [60]. This binding pattern contrasts sharply with canonical bacterial tetrameric SSB, where DNA wraps around the proteins [73]. DNA also wraps to several subunits of the trimeric archaeal-eucaryotic RPA [51, 74].

In this work, we explored the biological roles of OrfL and OrfM proteins encoded by ICE*St3*. As PrgE, they both harbour an OB-fold domain. Although very distant in primary sequence, they are homologous to HelP from ICE*Bs1* [18] and to Orf22 and Orf23 from Tn*916* [29]. HelP was shown to be essential for conjugative transfer. Since ICE*St3* encodes two OB-fold homologues, we initially hypothesised that one might, at least partially, complement the other, in which case neither would be essential for conjugation. Instead, our results showed that both *orfL* and *orfM* genes are essential for ICE*St3* conjugation. This suggests that each is responsible for distinct essential function(s) and/or that they must work together to ensure a common essential function required for DNA transfer. Supporting the second hypothesis, we demonstrated that OrfL and OrfM form a complex with a rather high affinity, using both *in vivo* and *in vitro* approaches (Fig. 2). Additionally, our BACTH analysis suggested OrfL could interact with RelSt3 and with the cellular PcrA helicase. Instead, this was not the case for OrfM, which only interacted with OrfL in our experiments. No interaction was detected between RelSt3 and PcrA. These findings suggest a networking role for OrfL, which could recruit the PcrA helicase to initiate RCR following RelSt3-mediated cleavage of the *oriT*, and thus might explain why it is strictly required for conjugation. Accordingly, the HelP protein from ICE*Bs1* was described as a helicase processivity helper [18]. Taken together, this point and our insights might support a general capacity of these OB-fold proteins to bind SF1 cellular helicases, even though no direct interaction was described between HelP and PcrA. Future work will be conducted to test whether OrfL/OrfM can, as demonstrated for HelP, support the processivity of a superfamily I helicase. Besides, ChIP-PCR experiments demonstrated that HelP was found associated with the ICE*Bs1 oriT* only in the presence of a catalytically active relaxase [18]. Indeed, HelP was no longer associated with *oriT* when the catalytic tyrosine of NicK was mutated. Thus, HelP could be recruited to *oriT* through interaction with the relaxase and/or through interaction with a specific conformation of the *oriT* DNA that would be generated only after relaxase nicking. EMSAs conducted with HelP would participate in resolving this question. Considering Orf22/Orf23 proteins from Tn*916*, pull-down assays demonstrated that they interact with their cognate Orf20 relaxase, but interaction with a cellular helicase was not tested [29]. As no biochemical data are available for HelP or Orf22/Orf23 proteins, their oligomerisation state is not yet known.

Both OrfM and OrfL were able to oligomerise partially as a dimeric form (Fig. 1). Consistently, they also both were shown to oligomerise in *E. coli* cells by BACTH approach (Fig. 2). This ability to oligomerise is reminiscent of PrgE behaviour, which oligomerises depending on salt and protein concentrations [60]. In pull-down assays, no direct interaction was observed between PrgE and its cognate MOB_P_ relaxase PcfG, nor with the auxiliary relaxosomal protein PcfF. However, weak interactions could have been revealed using more sensitive approaches or using *in cellulo* approaches such as BACTH. Importantly, Breidenstein *et al.* revealed that the *prgE* gene is not essential for pCF10 conjugation, whereas we demonstrated that both *orfL* and *orfM* genes are required for ICE*St3* conjugation. Notably, *prgE* gene is not localised in the vicinity of relaxosomal genes in pCF10 genome [60], whereas *orfL* and *orfM* genes are (Figure S4). All these data taken together highlight that OrfL/OrfM likely serve different function(s) in bacterial conjugation compared to PrgE. Whereas PrgE role could be focused on DNA protection as it was described for SSB encoded by the F family of enterobacterial plasmids, the OrfL/OrfM complex could play a more central role in DNA processing steps, potentially facilitating the rolling-circle replication.

The determination of the OrfM solution structure by NMR has highlighted several specificities relative to other OB-folds. Composed of 98 residues, OrfM can be considered as a small-to-medium-sized OB-fold, since sequence length ranges from 70 to 150 residues within this protein family [33]. OrfM has no capping helix between β_3_ and β_4_ nor long C-terminal extension. Apart from the canonical OB-fold β-barrel, the only additional secondary structure element is a helical turn at the N-terminus, while PrgE contains two helices in its extended L_34_ loop as well as a C-terminal helix [60]. PrgE has been noted to have a high content of helices compared to the canonical OB-fold, while this is not the case for OrfM. Besides, OrfM exhibits no very long β sheets protruding from the β-barrel, and it seems particularly dynamic outside its central core, which is consistent with the crystallisation difficulties encountered. The loop L_45_ has been shown to be particularly mobile; conformational exchange is mainly detected in the β-strand-like segment and is followed by an essentially unstructured loop. These mobile loops might adopt more structured conformations when in interaction with partners (i.e. DNA and/or relaxosomal proteins). The L_12_ loop also undergoes conformational exchange but remains globally more structured.

Dynamics is generally invoked in OB-fold crystallographic studies to explain the structural differences between the subunits of oligomeric OB-folds [75] or the absence of electron density. Few NMR relaxation studies have actually characterised the dynamics of OB-fold domains in solution. In SSB protein from *Sulfolobus solfataricus* [76], flexibility is observed in L_12_ but not in the L_45_ loop, which is much shorter than in OrfM structure. However, in the phage L capsid decoration protein, flexibility and conformational exchange are detected in L_45_ and L_23_ loops, as well as between β_3_ and the capping helix [77]. These observations suggest that the dynamic properties in OB-fold proteins can vary significantly, partly depending on the length of the loops.

Based on our results from various techniques, OrfM and OrfL are mainly found as monomeric but can also form dimers. As found for PrgE, their oligomeric state probably depends on various biophysical parameters such as salt concentration or pH. This suggests that the homodimer, when formed, may not be very tight. In contrast, experimental data indicated that OrfM strongly interacts with OrfL, and the AlphaFold model suggested that this interaction could occur via the N-terminal region (including an additional α_0_ helix turn) and the loop L_45_ of both proteins. This putative type of dimerisation resembles that found in many homodimeric OB-fold proteins such as *Neisseria gonorrhoeae* PriB (PDB 3K8A), whose residues of strand β_1_ form an intermolecular anti-parallel sheet [78]. This mode of association is also observed in the tetrameric *Mycobacterium leprae* SSB protein (Supplementary Fig. S5) [79]. Nevertheless, the corresponding region in OrfM unusually consists of a loop and an α-helix turn (residues 6–9) instead of a β-sheet. Interestingly, the dimeric AlphaFold models all exhibit a similar interface, whether for the putative homodimers (Orf23 and HelP) or for the heterodimers (OrfM/OrfL and Orf23/Orf22). These modelling results can be corroborated by the structure of the A3^OB^-A6 heterodimer obtained by co-crystallisation of two out of the six OB-fold domains of *Trypanosoma brucei* editosome (Supplementary Fig. S5) [80]. These two SSB proteins, sharing ~40% sequence identity, dimerise in a similar way as in the A6 homodimer, but the buried area is much larger and more intermolecular contacts are found, resulting in a tighter binding. According to AlphaFold model, OrfM and OrfL dimerise just like in this A3^OB^-A6 heterodimeric experimental structure. Moreover, our results also suggest that the OrfM–OrfL heterodimer is more stable than the homodimers. This might have biological consequences and be related to the intriguing existence of two different OB-fold proteins encoded by the same ICE (OrfM/OrfL from ICE*St3*, or Orf22/Orf23 from Tn*916*) in contrast to their single counterpart HelP from ICE*Bs1*. A search of OB-fold heterodimers also led us to identify the cases of *Thermoanaerobacter tengcongensis Tte*PriB and other thermostable SSBs, which all possess two consecutive OB-fold domains in the same chain [81]. The crystal structure of the N-terminal domain of *Tte*PriB is dimeric and the existence of a similar heterodimer with the C-terminal domain has been assumed on the basis of sequence alignment. However, the heterodimeric structure of the full-length protein has not been experimentally demonstrated.

A well-established role of auxiliary relaxosomal proteins corresponds to their involvement in *oriT* recognition and/or their regulation of the nicking-closing relaxase activities [8, 12, 82–84]. The fact that many OB-fold proteins are single-stranded binding proteins was another reason to investigate whether OrfL, OrfM, or the OrfL–OrfM complex were able to bind DNA. Interestingly, they all bind both ssDNA and dsDNA but with a very low affinity. Indeed, the addition of low concentrations of crosslinking agent (PFA) was required to visualise the binding by EMSA, whereas no PFA was required to demonstrate the interaction using the switchSENSE technology (Fig. 4). As the binding was not sequence specific, we ruled out the possibility that OrfL/OrfM could be involved in *oriT* recognition for the RelSt3 relaxase. This was not surprising, since we previously demonstrated that RelSt3 binds to the *oriT* through its N-terminal HTH domain, at a *bind* site located 68 bp downstream of the *nic* site [27]. This unusually large distance between the *bind* and the *nic* site would likely require the formation of a dsDNA loop upon interaction of RelSt3 with *oriT* [27], which could be favoured by the binding of OrfL/OrfM, especially given their demonstrated ability to bind dsDNA and ssDNA. This possibility will be addressed in future work. The literature on OB-fold complexes reveals that nucleic acids generally bind in the vicinity of the cleft formed by L_12_, β_2_, β_3_, and L_45_, notably through stacking interactions with aromatic sidechains or electrostatic interactions with positively charged residues [33]. Binding is mainly governed by electrostatic interactions in PriB protein, whereas stacking interactions are predominant in SSB proteins [81]. In OrfM the basic residues R26, K31, K34, R37, and H49 and the aromatic residues F27, F29, Y35, F72, and F90 could potentially participate in the interaction with DNA (Supplementary Fig. S8). Most of them are located in L_12,_ β_2_, and β_3_, with only F72 and F90 in L_45_. However, instead of protruding from the barrel, the loop L_12_, which contains two aromatics including F29 at the flexible tip of L_12_, folds down towards the bottom of the cleft, harbouring three other aromatics. This L_12_ loop tends to cover the cleft and incidentally bury some of the potential interacting residues (Supplementary Fig. S8), which could explain OrfM’s weak binding with nucleic acids. Bent conformations in L_12_ have also been observed, for instance, in *Sulfolobus solfataricus* SSB and human RPA-70 [75, 76, 85]; however, the tunnel in which nucleic acid has been shown to bind is wider than in OrfM (Supplementary Fig. S8). It can be noted that both jaws constituting the OB-fold clamp, L_12_ and especially L_45_, are dynamic in OrfM (Fig. 3B and C), and the resulting conformational fluctuations likely provide some versatility, enabling structural adjustments such as for DNA binding or for complex formation with OrfL.

## Supplementary Material

gkaf1161_Supplemental_Files

## Data Availability

Source data as well as plasmids and strains are available upon request. The NMR structure of OrfM is available under the PDB accession code 9HNJ.
