# Supplementary material for: Two OB-fold proteins from a Gram-positive conjugative element engage in relaxosome assembly and DNA processing
Source: Nucleic Acids Res. 2025 Nov 13;53(21):gkaf1161. doi: 10.1093/nar/gkaf1161 (PMC12614216; doi:10.1093/nar/gkaf1161)
Supplement: gkaf1161_Supplemental_Files [file gkaf1161_supplemental_files.zip › Laroussi et al - Revised Supplementary Material text-clean.docx]

**Supplementary Materials**

**Two OB-fold proteins from a Gram-positive conjugative element engage in relaxosome assembly and DNA processing**

Laroussi *et al*

**Supplementary figure legends**

**Figure S1. SEC analysis of an OrfL+OrfM mixture.** SEC analysis of a mixture of 30 µM OrfM with 15 µM OrfL with a Superdex S200 10/300 column (Cytiva). (**A**) Graph reporting absorbance at 280 nm (mAU) during elution as a function of elution volume (mL). Two major peaks of elution appeared with respective elution volumes of 14.45 and 17.50 mL. (**B**) SDS-PAGE analysis of the fractions corresponding to these two elution peaks. Both OrfL and OrfM proteins were present in the first peak (fractions 10-11) whereas only OrfM protein was detected in the second one (fractions 13-14).

**Figure S2: ^1^H-^15^N HSQC spectrum of OrfM** (0.545 mM) at 298 K in 20 mM HEPES buffer pH 7 with 100 mM NaCl and 10% D_2_O. Backbone amide signals are labelled with the residue number and « sc » refers to side-chain amide signals.

**Figure S3: AI (Artificial Intelligence) predicted structures of OrfM and OrfL (A, B), NMR (Nuclear Magnetic Resonance) ensemble of 20 structures of Sag0934 (C) and their comparison with the NMR structure of OrfM (D, E, F).** (**A**, **B**) The predicted structures are coloured according to the pLDDT confidence score (red to blue), with red indicating high-confidence regions. The pTM score of each predicted structure is given. (**C**) Sag0934 is a protein from *Streptococcus agalactiae*, which is identical to Orf22 of Tn*916* from *Enterococcus faecalis*. (**D**, **E**, **F**) Superimposition of OrfM NMR-structure with predicted structures of OrfM (**D**), OrfL (**E**) and with NMR structure of Sag0934 (**F**). The TM-score is given for each overlay. This score measures the structural similarity between two protein models and ranges from 0 to 1. Selected regions are labelled in all figures: canonical β-strands of the OB-fold (**β_1_**, **β_2_**, **β_3_**, **β_4_** and **β_5_**); canonical α-helix (**α_34_**) between **β_3_** and **β_4_** strands predicted only in OrfL; loops between **β_1_** and **β_2_** strands and **β_4_** and **β_5_** strands (**L_12_** and **L_45_**, respectively); N-terminal α-helix of OrfM (**α_0_**) putatively at the interface of the OrfM-OrfL heterodimer; N- and C-terminal ends of the models (**N_t_** and **C_t_**, respectively). Panels A, B, D and E provide an overview of regions that are similar or distinct when comparing experimental structures with AI-predicted models. Of particular note is the Nter of OrfM, observed rather unstructured by NMR except for the non-canonical α_0_ helical turn, while in the AlphaFold model it lies along β_4_ and the beginning of L_14_. Together with the loop L_45_, this N-terminal segment presents the highest RMSD values and the lowest pLDDT scores in the experimental and predicted structures, respectively. The additional helix turn α_0_ was also predicted in the OrfL model, though significant differences were found. AlphaFold predicted (i) an N-terminal extension of around 20 residues folded as a β-hairpin, (ii) an elongated L_12_ loop extended by about 20 residues, (iii) an α helix between the β_3_ and β_4_ strands (present in the canonical OB-fold), (iv) a rather structured β_4_-L_45_-β_5_ region with clearly elongated β_4_ and β_5_ strands, and (v) a C-terminal extension of about 15 residues, which was not confidently predicted.

**Figure S4: Genomic comparison of OB-fold encoding genes (A) and of OB-fold protein sequences (B,C) from ICE*St3*, Tn*916* and ICE*Bs1*.** (**A**) Sections of the genomes of ICE*St3* (green arrows), Tn*916* (blue arrows) and ICE*Bs1* (pink arrows) are represented to scale. *orfK*, *orf21* and *conQ* genes encode putative coupling proteins, whereas *orfJ*, *orf20* and *nicK* genes encode MOB_T_ relaxases of the respective ICEs. (**B**) Protein sequence alignements of OrfL, OrfM, Orf22, Orf23 and Help proteins and (**C**) corresponding percent identity matrix obtained with Clustal Omega sequence alignment software (1).

**Figure S5: AI (Artificial Intelligence) predicted structures of OrfM-OrfL heterodimer (A), Orf22-Orf23 heterodimer (B), Orf23 homodimer (C) HelP homodimer (D), and XRD (X-Ray Diffraction) structures of *Ml*SSB homotetramer (E) and of *Tb*A3OB-A6 heterodimer.** The ipTM score of each predicted structure is given. The resolution limit (*d_min_*) and R-factor (*Rf*) of each crystallographic structure is given. *Ml*SSB is a Single-Stranded DNA-Binding protein from *Mycobacterium leprae* (pdb entry 3AFP) and *Tb*A3OB-A6 is a complex of two editosome subunits from *Trypanosoma brucei* (OB-fold domain of subunit A3 and subunit A6, pdb entry 3STB). Selected regions are labelled in all figures.

**Figure S6: Binding isotherms and thermograms of OrfL, OrfM or OrfL-OrfM complex with RelSt3_64-410_ using ITC.** Experiments were performed at 298 K were obtained by injecting OrfL, OrfM or OrfL-OrfM complex at 142 µM into the shell containing RelSt3_64-410_ at 14 µM.

**Figure S7: OrfG and BSA proteins display no effect on RelSt3 endonuclease activity.** Nicking assays performed with the ori50 substrate and RelSt3 in presence of OrfG (**A**) or BSA (**B**). (**A**) Lane 1: ori50 substrate alone. Lanes 2 to 8: the same concentration of RelSt3 was used (3.2 µM). Increasing concentrations of OrfG were added as follows: lane 3, 4 µM; lane 4, 8 µM; lane 5, 12 µM; lane 6, 16 µM; lane 7, 24 µM; lane 8, 32 µM. (**B**) Lane 1: labelled 22 nt ssDNA alone as marker. Lane 2: ori50 substrate alone. Lanes 3 to 10: the same concentration of RelSt3 was used (3.2 µM). Increasing concentrations of BSA were added as follows: lane 4, 2 µM; lane 5, 4 µM; lane 6, 8 µM; lane 7, 12 µM; lane 8, 16 µM; lane 9, 24 µM; lane 9, 32 µM.

**Figure S8: Structural comparison of three OB-fold domains**: (**A**) OrfM (this work); (**B**) SSB protein from *Sulfolobus solfataricus* (PDB code 1O7I); (**C**) domain A of human RPA70-A (PDB code 1JMC). Aromatic and positively charged residues in the OB-fold cleft are represented in brown and blue, respectively. Those in bold characters (in *Sso*SSB and hRPA70) have been shown to be involved in DNA binding (2, 3).

**Supplementary tables**

**Table S1. Bacterial strains used in this work**

| **Strains** | **Genotype or description** | **Source or Reference** |
| --- | --- | --- |
| ***E. coli* strains** | | |
| EC101 | *supE hsd-5 thi* (*lac-proAB*) F (*traD6 proAB lacI*^q^ *lacZ* M15) repA, derivative of strain TG1 (56) *repA*, derivative of strain JM101 | Laboratory stock, (4) |
| DH5α | F^-^ Φ80*lac*ZΔM15 Δ(*lac*ZYA-*arg*F) U169 *rec*A1 *end*A1 *hsd*R17(r_k_^-^, m_k_^+^) *pho*A *sup*E44 *thi*-1 *gyr*A96 *rel*A1 λ^-^ | Invitrogen |
| BL21(DE3) | str. B F^–^ *ompT* *gal* *dcm* *lon* *hsdS_B_*(*r_B_*^–^*m_B_*^–^) λ(DE3 [*lacI* *lacUV5*-*T7p07* *ind1* *sam7* *nin5*]) [*malB*^+^]_K-12_(λ^S^) | Laboratory stock, (5) |
| BL21 (DE3)-pSKB3-*orfL* | BL21 strain producing OrfL from ICE*St3* | This work |
| BL21 (DE3)-pSKB3-*orfM* | BL21 strain producing OrfM from ICE*St3* | This work |
| ***S. thermophilus* strains** | | |
| LMG18311 (ICE*St3cat*) | LMG18311 strain carrying ICE*St3* tagged with the *cat* gene inserted in the pseudogene *Ψorf385J*, Cm^r^ | (6) |
| LMG18311 (ICE*St3ΔorfLcat*) | LMG18311strain carrying ICE*St3* deleted for the orfL gene, and tagged with the *cat* gene inserted in the pseudogene *Ψorf385J*, Cm^r^ | This work |
| LMG18311 (ICE*St3ΔorfMcat*) | LMG18311strain carrying ICE*St3* deleted for the orfM gene, and tagged with the *cat* gene inserted in the pseudogene *Ψorf385J*, Cm^r^ | This work |
| LMG18311 (pMG36e) | LMG18311 carrying pMG36e, a plasmid conferring erythromycin resistance | (6) |
| LMG18311 (ICESt3*ΔorfLcat+orfL)* | LMG18311 (ICE*St3ΔorfLcat*) complemented *in trans* by *orfL* gene | This work |
| LMG18311 (ICESt3*ΔorfMcat+orfM)* | LMG18311 (ICE*St3ΔorfMcat*) complemented *in trans* by *orfM* gene | This work |

**Table S2. Plasmids used in this work**

| **Plasmid** | **Description** | **Source or Reference** |
| --- | --- | --- |
| pTH24 | His-tagged-TEV protease coding sequence under control of T7/lac promoter | (7) |
| pSKB3 | Expression vector derived from pET28a including an in frame 6 His-tag and a TEV protease cleavage site in the N-terminal sequence | Gift from Stephen K. Burley |
| pSKB3::*orfL* | *orfL* cloned in frame of 6His-TEV in pSKB3 | This work |
| pSKB3::*orfM* | *orfM* cloned in frame of 6His-TEV in pSKB3 | This work |
| pSKB3::*orfJ* | *orfJ* (encoding RelSt3) cloned in frame of 6His-TEV in pSKB3 | (8) |
| pG^+^Host9 | 3.8 kb, pWV01-type thermosensitive replication origin from pVE6002, Erm^r^ | (9) |
| pG^+^host9*ΔorfL* | pG^+^Host9 carrying the 1072 bp upstream region of *orfL* CDS and the *orfM* 1033 bp downstream region of *orfL* CDS, Erm^r^ | This work |
| pG^+^host9*ΔorfM* | pG^+^Host9 carrying the 1093 bp upstream region of *orfM* CDS and the *orfM* 1117 bp downstream region of *orfL* CDS, Erm^r^ | This work |
| pMG36e | 3.4 kb, replication origin from pWV01, Ery^r^ | (10) |
| pBR322-*oriT* | pBR322 plasmid with the intergenic *orfJ*/*orfK* region (including *oriT nic* sequence) of ICE*St3* cloned between *Eco*RI and *Hind*III restriction sites | (8) |
| 18-Pal | *pal* cloned downstream T18 into pEB355 | (11) |
| 25-TolB | *tolB* cloned upstream T25 into pEB354 | (11) |
| pUT18C | High copy number vector that encodes the T18 fragment that is fused upstream of the MCS. This vector allows creating in-frame fusions at the C-terminal end of T18. Ampicillin resistance | Euromedex |
| pUT18 | High copy number vector that encodes the T18 fragment that is fused downstream of the MCS. This vector allows creating inframe fusions at the N-terminal end of T18. Ampicillin resistance | Euromedex |
| pKT25 | Low copy number vector that encodes the T25 fragment that is fused upstream of the MCS. This vector allows creating in-frame fusions at the C-terminal end of T25. Ampicillin resistance. | Euromedex |
| pKTN25 | Low copy number vector that encodes the T25 fragment that is fused downstream of the MCS. This vector allows creating inframe fusions at the N-terminal end of T25. Ampicillin resistance. | Euromedex |
| pUT18C-RelSt3 | *OrfJ* cloned downstream the T18 in pUT18C | This work |
| pKT25-RelSt3 | *OrfJ* cloned downstream the T25 in pKT25 | This work |
| pUT18C-*OrfL* | *OrfL* cloned downstream the T18 in pUT18C | This work |
| pKT25-*OrfL* | *OrfL* cloned downstream the T25 in pKT25 | This work |
| pUT18C-*OrfM* | *OrfM* cloned downstream the T18 in pUT18C | This work |
| pKT25-*OrfM* | *OrfM* cloned downstream the T25 in pKT25 | This work |
| pUT18C-*PcrA* | *PcrA* from *S. thermophilus* LMG18311 cloned downstream the T18 in pUT18C | This work |
| pKT25-*PcrA* | *PcrA* cloned downstream the T25 in pKT25 | This work |
| pUT18-RelSt3 | *OrfJ* cloned upstream the T18 in pUT18C | This work |
| pKTN25-RelSt3t | *OrfJ* cloned upstream the T25 in pUT18C | This work |
| pUT18-*OrfL* | *OrfL* cloned upstream the T18 in pUT18C | This work |
| pKTN25-*OrfL* | *OrfL* cloned upstream the T25 in pUT18C | This work |
| pUT18-*OrfM* | *OrfM* cloned upstream the T18 in pUT18C | This work |
| pKTN25-*OrfM* | *OrfM* cloned upstream the T25 in pUT18C | This work |
| pUT18-*PcrA* | *PcrA* cloned upstream the T18 in pUT18C | This work |
| pKTN25-*PcrA* | *PcrA* cloned upstream the T25 in pUT18C | This work |

**Table S3. Oligonucleotides used in this work**

All oligonucleotides were purchased from Eurogentec, France. Restriction sites are underlined, and initiator codons are indicated with bold characters.

| **Name** | **Sequence** |  |
| --- | --- | --- |
| **Oligonucleotides used for deletion of the *orfL* gene in ICE*St3*** | | |
| I∆L1_For | gggcggccgcGTCAAGTCCGTTTATGGTTG |  |
| I∆L2_Rev | CATATGTATTTCTCCTTTACTTTCTTA |  |
| II∆L3_For | GTAAAGGAGAAATACATATGAAGCTCTTTACCTATCGTGG |  |
| II∆L4_Rev | cggggcccATAGTCACGGTCAATCTTGG |  |
| **Oligonucleotides used for deletion of the *orfM* gene in ICE*St3*** | | |
| I∆M1_For | gggcggccgcAAAATTTGTGCAATCCTGAC |  |
| I∆M2_Rev | CATAGAATTTTCCTTTCTAAATTTAAG |  |
| II∆M3_For | AGAAAGGAAAATTCTATGGCTAAAATCGGATTAAACTACG |  |
| II∆M4_Rev | cggggcccATAGTCCCAAGTGACGTCTG |  |
| **Oligonucleotides used for construction of complemented strain with *orfL* or *orfM* genes *in trans* at locus *tRNASer*** | | |
| tRNASerFor | GACGATCTCTTAGCAGGTAT |  |
| tRNASerRev | ACCTCAGGATTGATTTATTTGG |  |
| PtetATGrev | CATTATTTTTCCTCCTTATTTATTTAGATC |  |
| ATGhlpA | ATGGCAAACAAACAAGATTTGA |  |
| PtetATGorfL-For | GATCTAAATAAATAAGGAGGAAAAATAATGGCTAAAATCGGATTAAACTACGG |  |
| OrfLRBSHplA-Rev | TTTAGCGATCAAATCTTGTTTGTTTGCCATAGAGTTCTCCTAATTATCTTTCTTGTTGTC |  |
| PtetATGorfM-For | GATCTAAATAAATAAGGAGGAAAAATAATGATTCCAGAAACACTTGTTATGA |  |
| OrfMRBSHplA-Rev | TTTAGCGATCAAATCTTGTTTGTTTGCCATAGAGTTCTCTTATTTTTTTAGGACTAATTTTTCCGC |  |
| **Oligonucleotides used for cloning of *orfL* or *orfM* CDS segments into pSKB3 for over-expression in *E. coli*** | | |
| OrfL-For | CCAAACATATGGCTAAAATCGGATTAAAC |  |
| OrfL-Rev | GGAAAAAGCTTCTAATTATCTTTCTTGTTGTCTAGAG |  |
| OrfM-For | CCAAACATATGATTCCAGAAACACTTGTTATG |  |
| OrfM-Rev | GGAAACAAGCTTTTATTTTTTTAGGACTAATTTTTCCG |  |
|  |  |  |
| **Oligonucleotides used for cloning of *orfL*, *orfM*, *orfJ* (RelSt3) or *pcrA* (PcrA from *S. thermophilus*) genes in BACTH vectors** | | |
| T25-OrfL-For | AGCAACTGCAGGTGCTAAAATCGGATTAAACTAC |  |
| T18-OrfL-For | AGCAACTGCAGTGCTAAAATCGGATTAAACTAC |  |
| OrfL-T25/T18-For | AGCAAAAGCTTAGCTAAAATCGGATTAAACTAC |  |
| T25/T18-OrfL-Rev | AGCAACCCGGGCTAATTATCTTTCTTGTTGTCTAGAG |  |
| OrfL-T25/T18-Rev | AGCAACCCGGGCTAATTATCTTTCTTGTTGTCTAGAG |  |
| T25-OrfM-For | AGCAACTGCAGGTATTCCAGAAACACTTGTTATG |  |
| T18-OrfM-For | AGCAACTGCAGTATTCCAGAAACACTTGTTATG |  |
| OrfM-T25/T18-For | AGCAAAAGCTTAATTCCAGAAACACTTGTTATG |  |
| T25/T18-OrfM-Rev | AGCAACCCGGGTTATTTTTTTAGGACTAATTTTTCCGC |  |
| OrfM-T25/T18-Rev | AGCAACCCGGGTTTTTTTTAGGACTAATTTTTCCGC |  |
| T25-RelSt3-For | AGCAACTGCAGGTACTAAAATAAGTCCCTTTCAG |  |
| T18- RelSt3-For | AGCAACTGCAGTACTAAAATAAGTCCCTTTCAG |  |
| RelSt3-T25/T18-For | AGCAAAAGCTTAACTAAAATAAGTCCCTTTCAG |  |
| T25/T18- RelSt3-Rev | AGCAACCCGGGCTAGTGTTCATAGTGTTTGTTGG |  |
| RelSt3-T25/T18-Rev | AGCAACCCGGGTGTGTTCATAGTGTTTGTTGG |  |
| T25-PcrA -For | AGCAACTGCAGGTAACCCATTATTAACTGGTATGAATG |  |
| PcrA -T25/T18-For | AGCAACTGCAGTAACCCATTATTAACTGGTATGAATG |  |
| T25/T18- PcrA-Rev | AGCAACCCGGGCTATTTCTTCACAATAGGTGCTAC |  |
| PcrA-T25/T18-Rev | AGCAACCCGGGTTTTCTTCACAATAGGTGCTAC |  |

**Oligonucleotide used in activity assays**

| \| 22 nt marker \| GAGACTTCAACCCCCGATTTCT \| \| --- \| --- \| \| ori50 \| GACTTCAACCCCCGATTTCTAATAGGGGGGTTACATTTTCAAGATTTAGAAAGTGTGTCACTTTGGTCCA AAAAGTGTGTCACTTAGTCAAAAGGAG \| \| ori57 \| AGGCGCAGACCGTAGCCCGAAGTTCCTAGGCCATGATGAGACTTCAACCCCCGATTTCT \| \| ori41C \| CTCCTTTTGACTAAGTGACACACTTTTTGGACCAAAGTGACACACTTTCTAAATCTTGA \| |
| --- | --- | --- | --- | --- | --- | --- | --- | --- |

**Oligonucleotide used for the switchSENSE assays**

| \| ssDNA-overhang \| GAGACTTCAACCCCCGATTTCTAATAGGGGGGTTACATTTTCAAGATTTAGAAAGTGTGTCACTTTGGTCCAAAAAGTGTGTCACTTAGTCAAAAGGAGATATAT**CAGCGTTCGATGCTTCC GACTAATCAGCCATATCAGCTTACGACTA**  Oligonucleotide composed by the complementary sequence of the Nanolever (in bold) and the single strand DNA sequence (ssDNA) originated from ICE*St3* (underlined) \| \| --- \| --- \| \| c-DNA \| CTCCTTTTGACTAAGTGACACACTTTTTGGACCAAAGTGACACACTTTCTAAATCTTGAAAATGTAACCCCCCTATTAGAAATCGGGGGTTGAAGTCTC  Oligonucleotide corresponding to the complementary sequence of ssDNA (sequence underlined in ssDNA-overhang) originated from ICE*St3* in order to create a dsDNA-overhang  This sequence is also used for chemical crosslinking *in vitro* in order to create a dsDNA \| \|  \|  \| |
| --- | --- | --- | --- | --- | --- | --- |

**Oligonucleotides used for EMSA**

| \| ssDNA \| GAGACTTCAACCCCCGATTTCTAATAGGGGGGTTACATTTTCAAGATTTAGAAAGTGTGTC ACTTTGGTCCAAAAAGTGTGTCACTTAGTCA AAAGGAG  Sequence corresponding to the ssDNA fragment used for switchSENSE experiment labeled with 6-FAM at 5’ end for EMSAs. \| \| --- \| --- \| |
| --- | --- | --- |

**Table S4. Statistics for the ensemble of 10 NMR models of OrfM**

| Description | Value |
| --- | --- |
| Total NOE restraints | 679 |
| Intra-residue (\|i-j\|= 0) | 243 |
| Sequential (\|i-j\|=1) | 230 |
| Medium range (1<\|i-j\|<5) | 49 |
| Long range (\|i-j\|≥5) | 157 |
| H-bond restraints | 25 |
| Dihedral restraints ^(a)^ | 74 |
| Average number of NOE violations |  |
| 0.2-0.5 Å | 31 |
| > 0.5 Å | 30 |
| Clashscore | 7 |
| Ramachandran outliers (%) | 3.5 |
| Sidechain outliers (%) | 5.0 |
| RMSD (Å) ^(b)^ |  |
| Backbone | 0.88 ± 0.17 |
| Heavy atoms | 1.77 ± 0.22 |

^(a)^ Dihedral restraints were generated using DANGLE.

^(b)^ RMSD were calculated over residues 15-26, 33-72, 87-97

**Table S5. Oligomer predictions using AlphaFold 3^1^**

|  | **ipTM score** | |
| --- | --- | --- |
| Description^2^ | **Dimer** | **Tetramer^3^** |
| OrfM-OrfL | 0 .88 | 0.64^4^ |
| OrfL-OrfL | 0.17 | 0.16 |
| OrfM-OrfM | 0.18 | 0.21 |
| HelP-HelP | 0.68 | 0.70 |
| Orf22-Orf22 | 0.36 | 0.21 |
| Orf23-Orf23 | 0.82 | 0.29 |
| Orf23-Orf22 | 0.85 | 0.52^4^ |

(1) Reference (12); (2) The references for the sequences used to predict the dimers and tetramers are as follows: OrfM GenBank CAE52359.1, OrfL NCBI WP_070654566.1, HelP NCBI WP_063335935.1, Orf22 GenBank HFH9489047.1, and Orf23 GenBank EPT94776.1; (3) None of the predicted tetramers display the arrangement observed in the tetrameric crystal structure of the single-stranded DNA-binding protein from *Mycobacterium leprae* (see Figure S5E); (4) The stoichiometry of the predicted heterotetramer is 2:2.

#### Supplementary References

1. Sievers,F., Wilm,A., Dineen,D., Gibson,T.J., Karplus,K., Li,W., Lopez,R., McWilliam,H., Remmert,M., Söding,J., *et al.* (2011) Fast, scalable generation of high‐quality protein multiple sequence alignments using Clustal Omega. *Molecular Systems Biology*, **7**, 539.

2. Kerr,I.D., Wadsworth,R.I.M., Cubeddu,L., Blankenfeldt,W., Naismith,J.H. and White,M.F. (2003) Insights into ssDNA recognition by the OB fold from a structural and thermodynamic study of Sulfolobus SSB protein. *The EMBO Journal*, **22**, 2561–2570.

3. Bochkarev,A., Pfuetzner,R.A., Edwards,A.M. and Frappier,L. (1997) Structure of the single-stranded-DNA-binding domain of replication protein A bound to DNA. *Nature*, **385**, 176–181.

4. Leenhouts,K. (1995) Integration strategies and vectors. *Developments in biological standardization*, **85**, 523–530.

5. Studier,F.W. and Moffatt,B.A. (1986) Use of bacteriophage T7 RNA polymerase to direct selective high-level expression of cloned genes. *Journal of molecular biology*, **189**, 113–130.

6. Bellanger,X., Roberts,A.P., Morel,C., Choulet,F., Pavlovic,G., Mullany,P., Decaris,B. and Guédon,G. (2009) Conjugative transfer of the Integrative Conjugative Elements ICE*St1* and ICE*St3* from *Streptococcus thermophilus*. *Journal of Bacteriology*, **191**, 2764–2775.

7. van den Berg,S., Löfdahl,P.-Å., Härd,T. and Berglund,H. (2006) Improved solubility of TEV protease by directed evolution. *Journal of Biotechnology*, **121**, 291–298.

8. Soler,N., Robert,E., Chauvot de Beauchêne,I., Monteiro,P., Libante,V., Maigret,B., Staub,J., Ritchie,D.W., Guédon,G., Payot,S., *et al.* (2019) Characterization of a relaxase belonging to the MOBT family, a widespread family in Firmicutes mediating the transfer of ICEs. *Mob. DNA*, **10**, 10.1186.

9. Maguin,E., Duwat,P., Hege,T., Ehrlich,D. and Gruss,A. (1992) New thermosensitive plasmid for gram-positive bacteria. *Journal of bacteriology*, **174**, 5633–5638.

10. van de Guchte,M., Van der Vossen,J.M., Kok,J. and Venema,G. (1989) Construction of a lactococcal expression vector: expression of hen egg white lysozyme in *Lactococcus lactis* subsp. *lactis*. *Applied and Environmental Microbiology*, **55**, 224–228.

11. Battesti,A. and Bouveret,E. (2012) The bacterial two-hybrid system based on adenylate cyclase reconstitution in *Escherichia coli*. *Methods*, **58**, 325–334.

12. Abramson,J., Adler,J., Dunger,J., Evans,R., Green,T., Pritzel,A., Ronneberger,O., Willmore,L., Ballard,A.J., Bambrick,J., *et al.* (2024) Accurate structure prediction of biomolecular interactions with AlphaFold 3. *Nature*, **630**, 493–500.
