# Supplementary material for: Two OB-fold proteins from a Gram-positive conjugative element engage in relaxosome assembly and DNA processing
Source: Nucleic Acids Res. 2025 Nov 13;53(21):gkaf1161. doi: 10.1093/nar/gkaf1161 (PMC12614216; doi:10.1093/nar/gkaf1161)
Supplement: gkaf1161_Supplemental_Files [file gkaf1161_supplemental_files.zip › Laroussi et al - Supplementary Figures.pptx]

## Slide 1
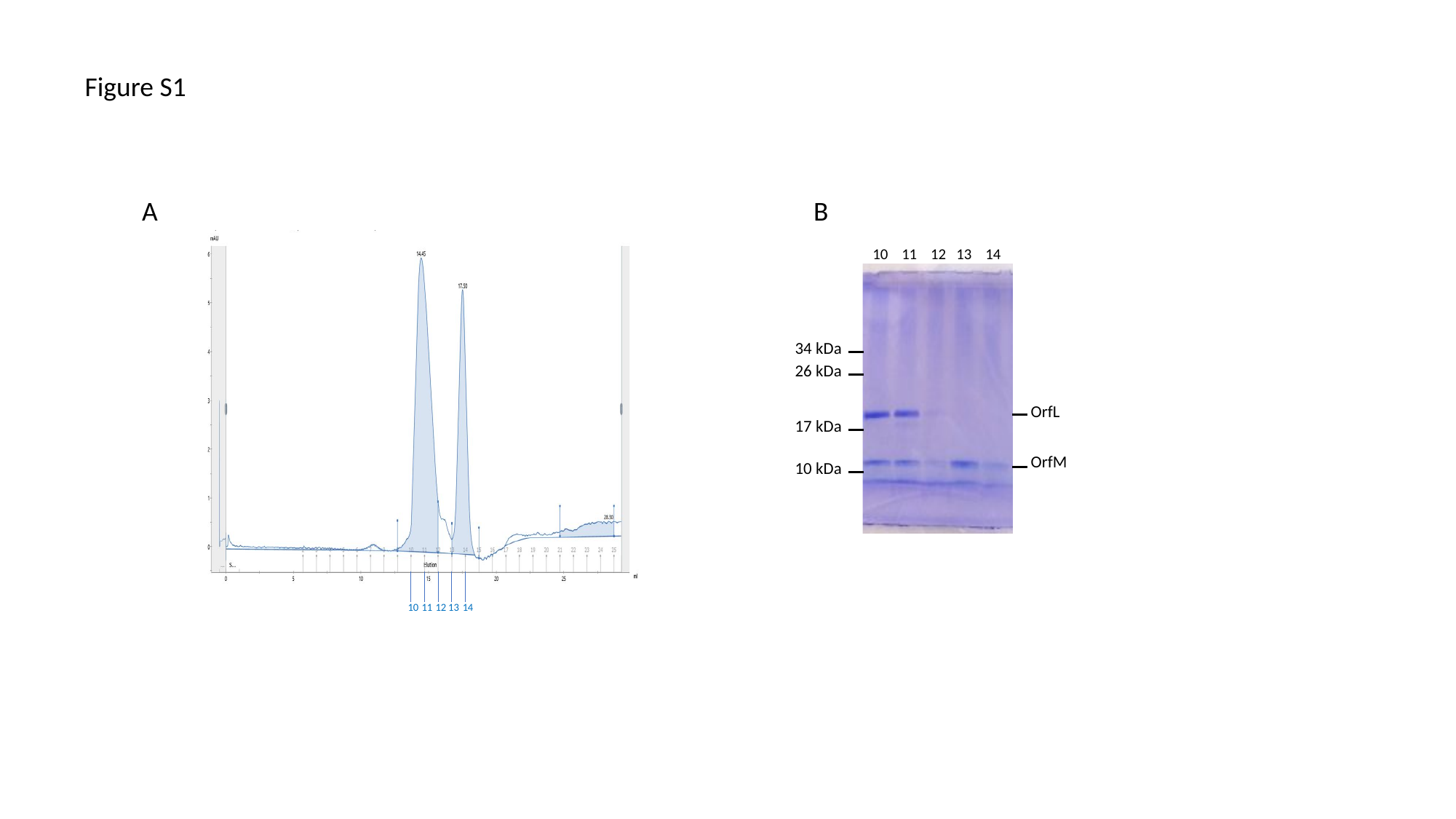

Figure S1
A
B
10 11 12 13 14
34 kDa
26 kDa
OrfL
17 kDa
OrfM
10 kDa
10
11
12
13
14

## Slide 2
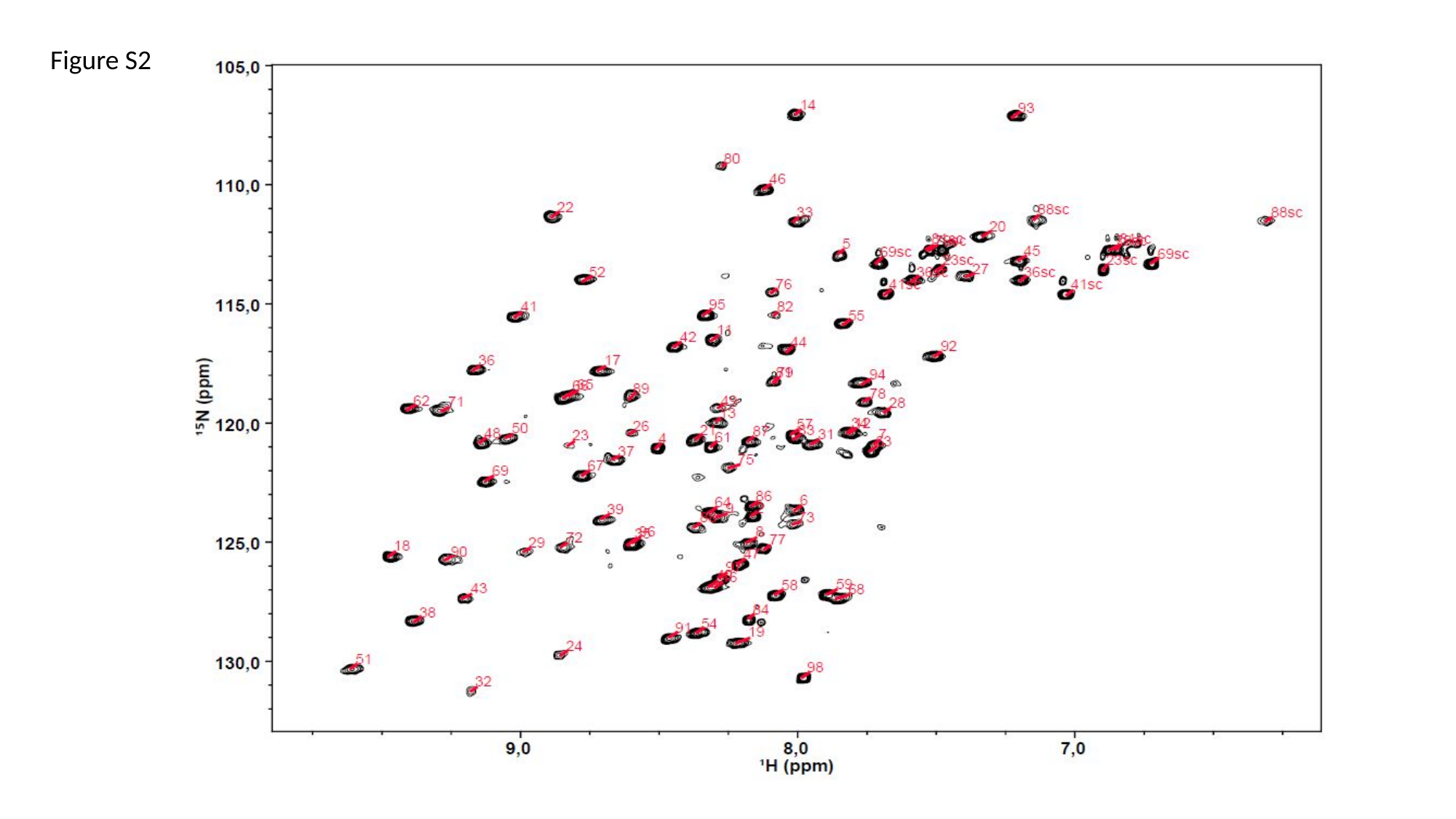

Figure S2

## Slide 3
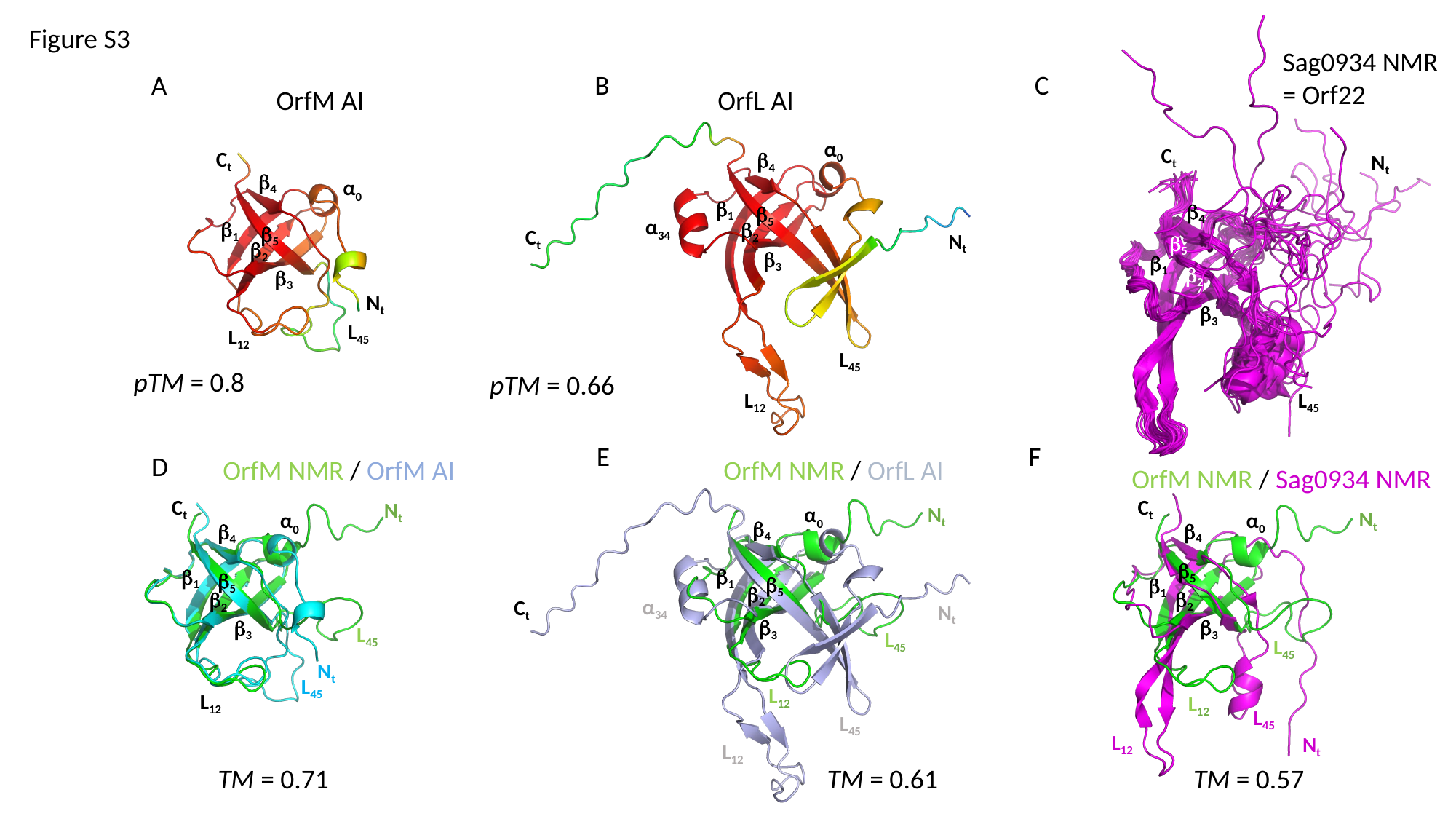

Figure S3
Sag0934 NMR
= Orf22
A
B
C
OrfM AI
OrfL AI
α0
Ct
Ct
β4
Nt
β4
α0
β1
β4
β5
α34
β1
β2
β5
Ct
Nt
β5
β2
β3
β1
β2
β3
Nt
β3
L45
L12
L45
pTM = 0.8
pTM = 0.66
L45
L12
F
E
D
OrfM NMR / OrfM AI
OrfM NMR / OrfL AI
OrfM NMR / Sag0934 NMR
Ct
Ct
Nt
α0
Nt
Nt
α0
α0
β4
β4
β4
β5
β1
β1
β5
β5
β1
β2
β2
β2
α34
Ct
Nt
β3
β3
β3
L45
L45
L45
Nt
L45
L12
L12
L12
L45
L45
L12
Nt
L12
TM = 0.71
TM = 0.61
TM = 0.57

## Slide 4
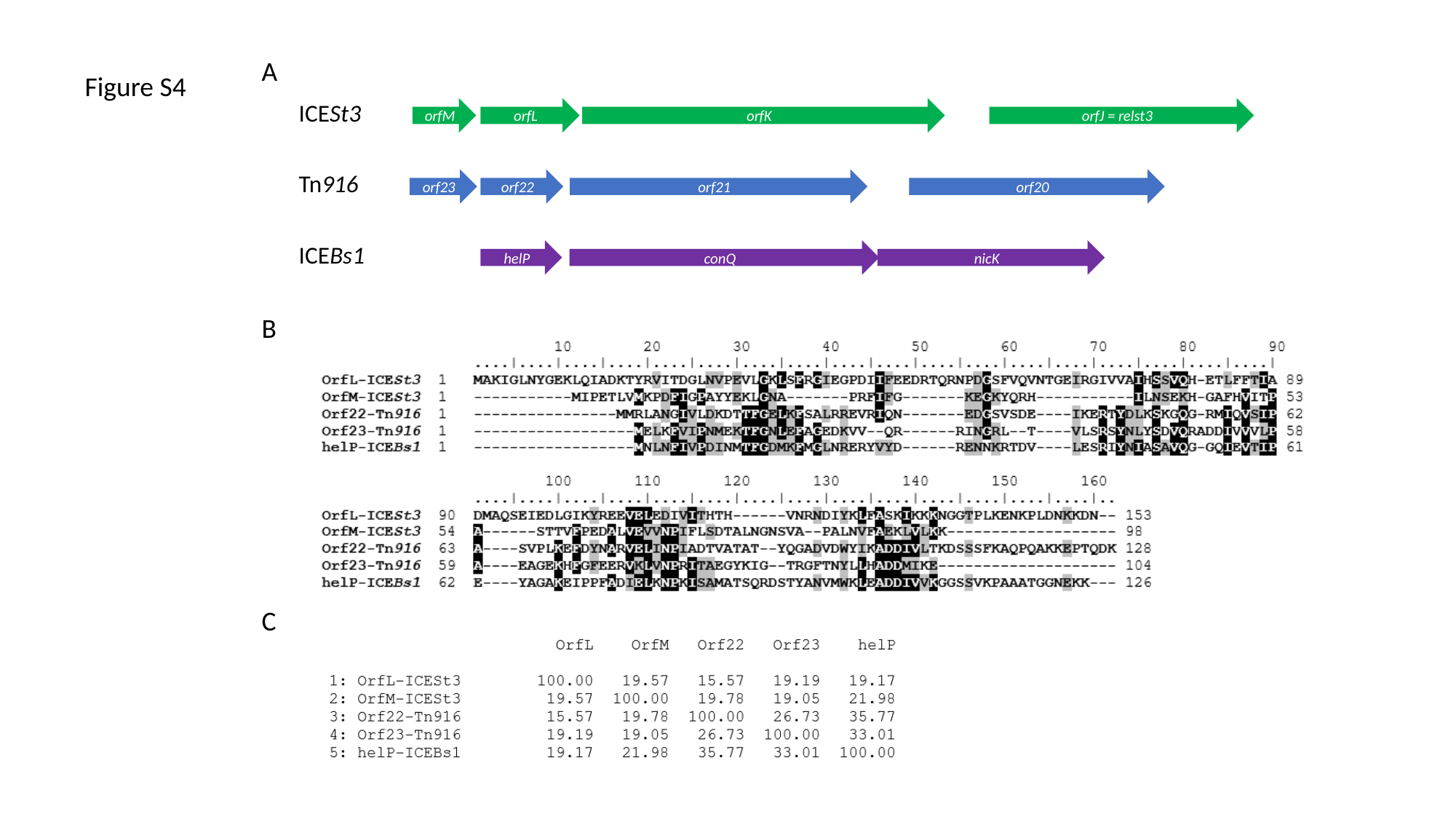

A
Figure S4
ICESt3
orfM
orfL
orfK
orfJ = relst3
Tn916
orf23
orf22
orf21
orf20
ICEBs1
helP
conQ
nicK
B
C

## Slide 5
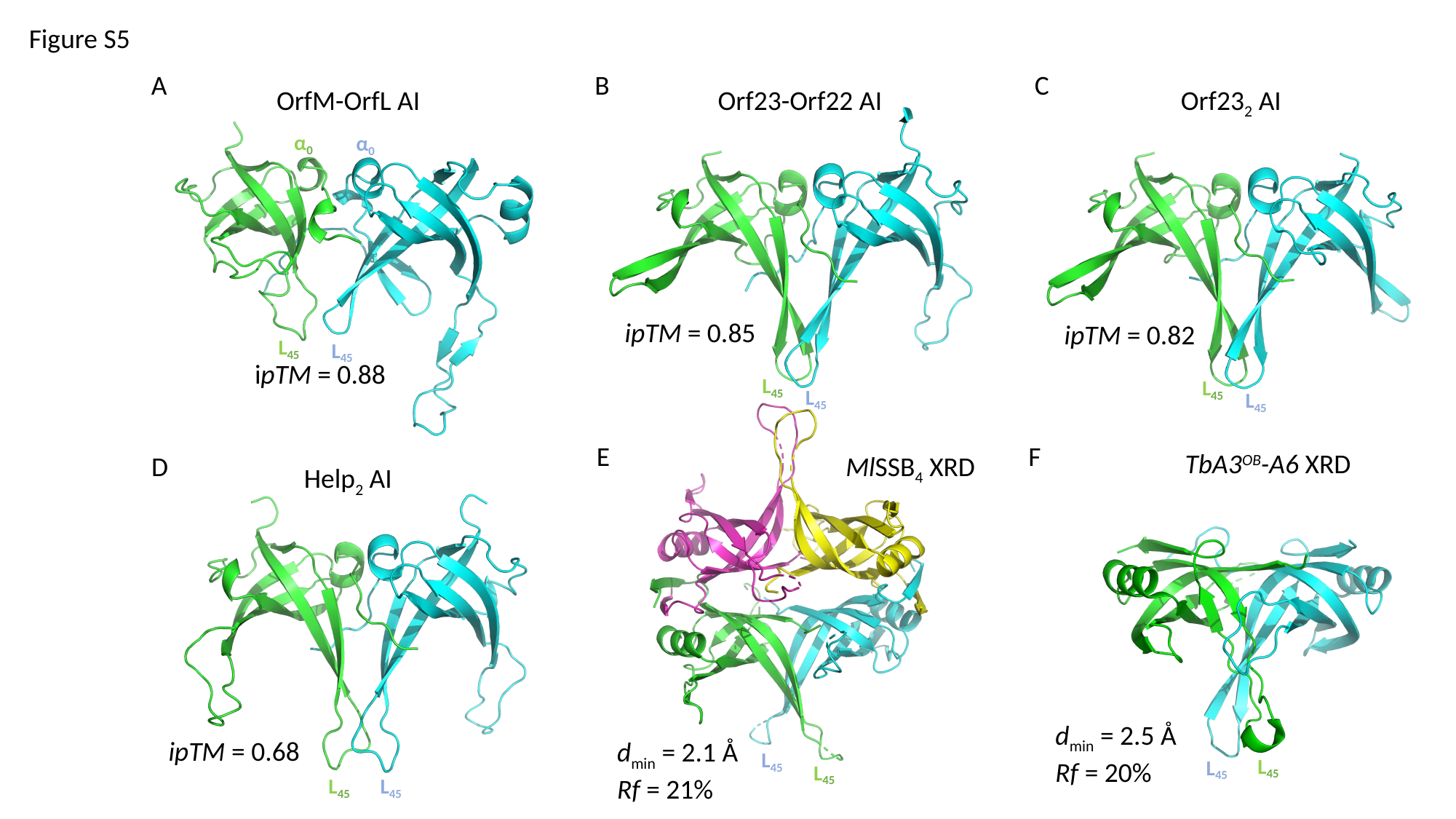

Figure S5
A
B
C
OrfM-OrfL AI
Orf23-Orf22 AI
Orf232 AI
α0
α0
ipTM = 0.85
ipTM = 0.82
L45
L45
ipTM = 0.88
L45
L45
L45
L45
L45
F
E
TbA3OB-A6 XRD
D
MlSSB4 XRD
Help2 AI
dmin = 2.5 Å
Rf = 20%
ipTM = 0.68
dmin = 2.1 Å
Rf = 21%
L45
L45
L45
L45
L45
L45

## Slide 6
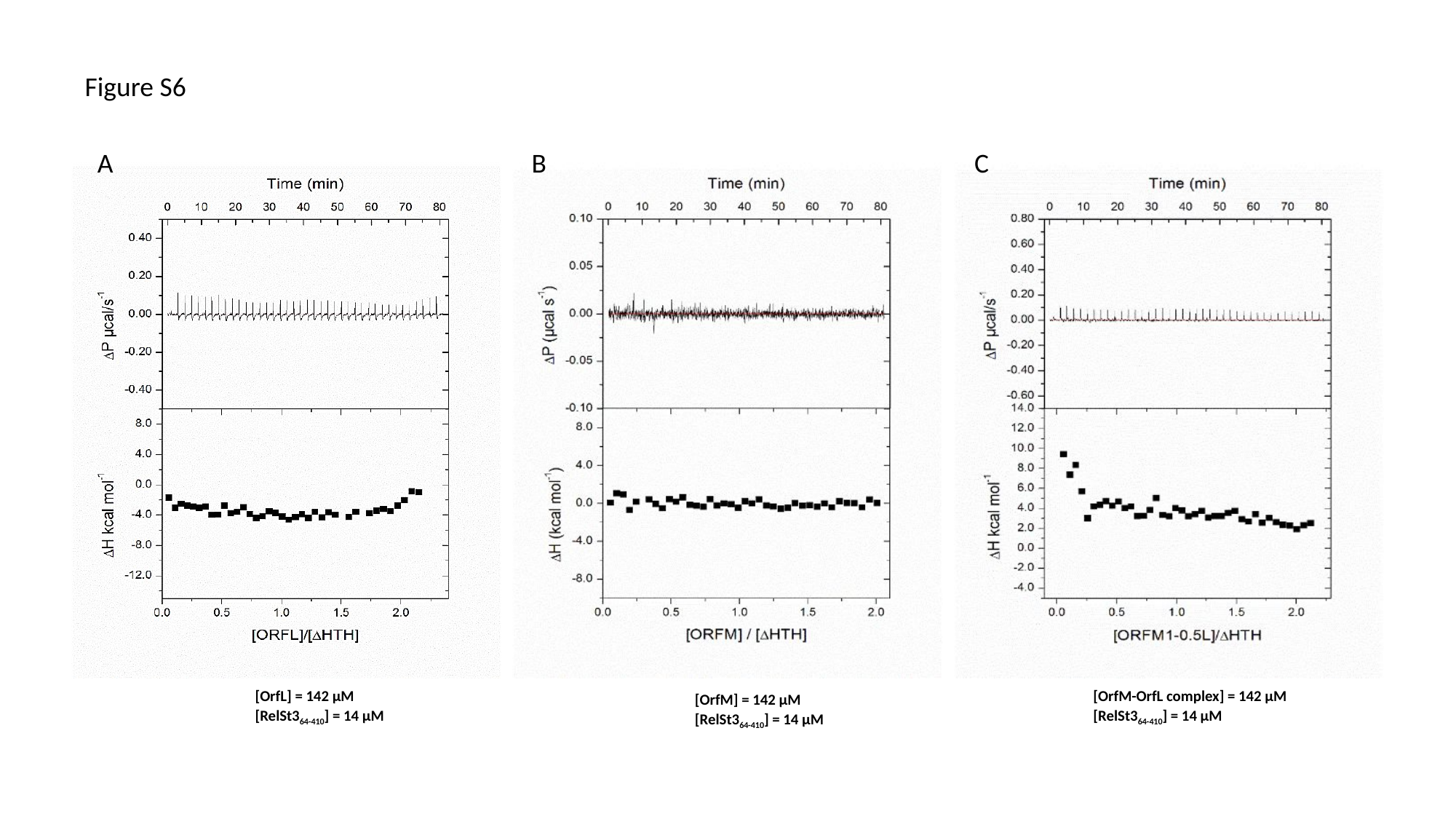

Figure S6
C
A
B
[OrfL] = 142 µM [RelSt364-410] = 14 µM
[OrfM-OrfL complex] = 142 µM [RelSt364-410] = 14 µM
[OrfM] = 142 µM [RelSt364-410] = 14 µM

## Slide 7
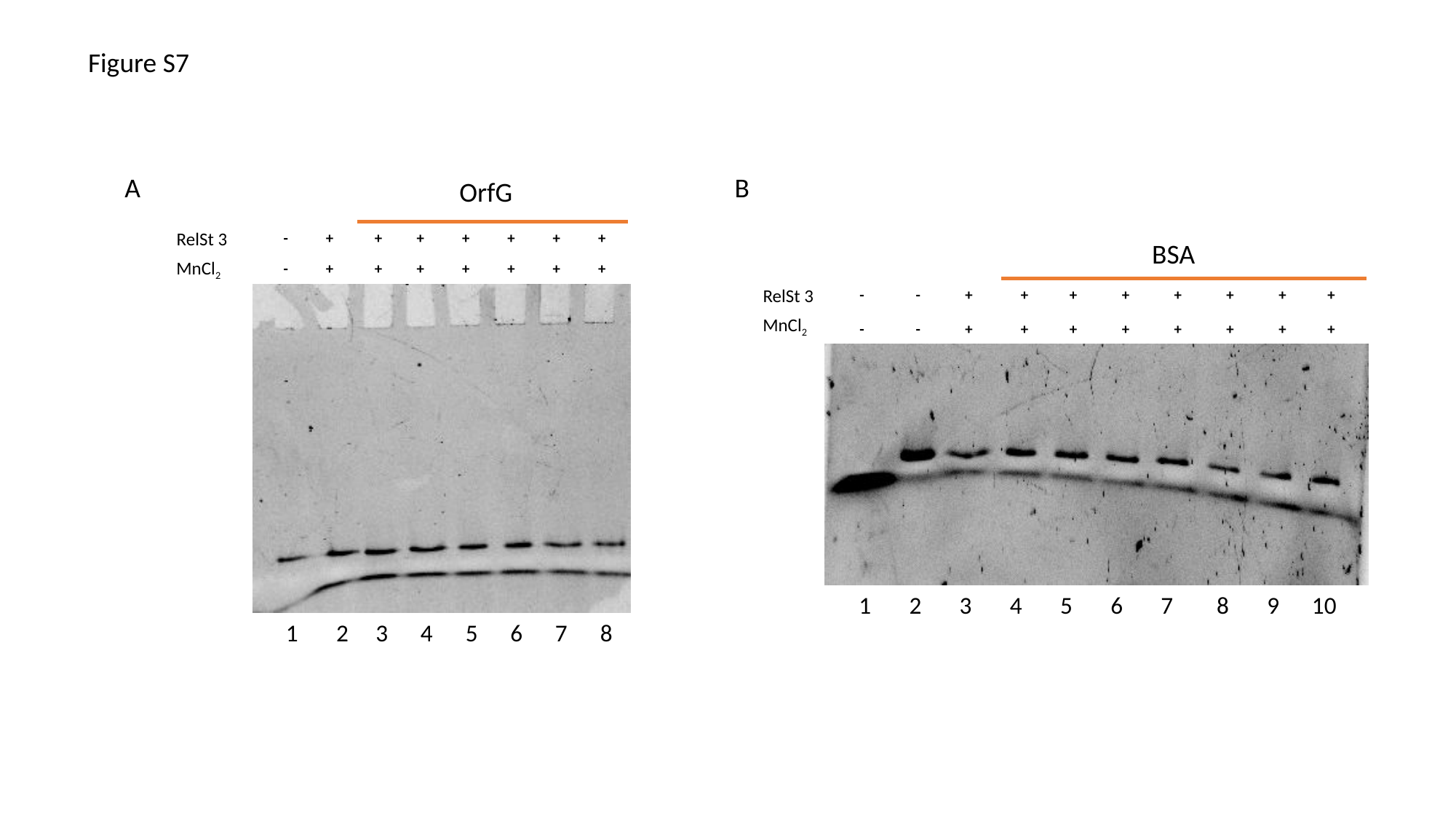

Figure S7
A
B
OrfG
RelSt 3
- + + + + + + +
BSA
MnCl2
- + + + + + + +
RelSt 3
 - - + + + + + + + +
MnCl2
 - - + + + + + + + +
1 2 3 4 5 6 7 8 9 10
1 2 3 4 5 6 7 8

## Slide 8
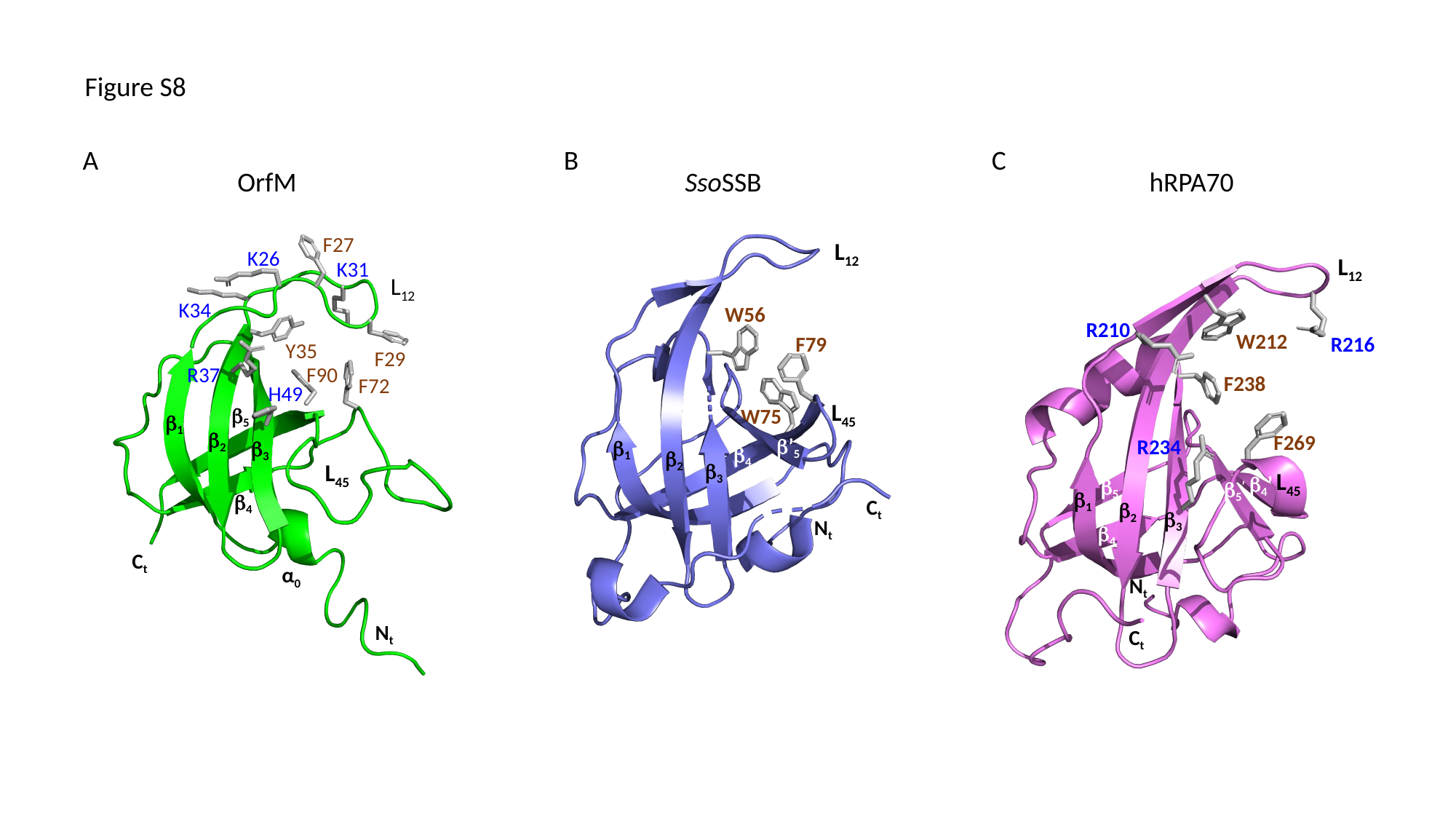

Figure S8
A
B
C
OrfM
SsoSSB
hRPA70
F27
L12
K26
L12
K31
L12
K34
W56
R210
W212
R216
 F79
Y35
F29
R37
F90
F238
 F72
H49
L45
β5
W75
β1
β2
F269
β’5
R234
β1
β3
β4
β2
L45
β3
L45
β4’
β5
β5’
β1
β4
Ct
β2
β3
Nt
β4
Ct
α0
Nt
Nt
Ct
